# Identification, molecular evolution, codon bias, and expansion analysis of NLP transcription factor family in foxtail millet (*Setaria italica* L.) and closely related crops

**DOI:** 10.3389/fgene.2024.1395224

**Published:** 2024-05-21

**Authors:** Huilong Chen, Fang Liu, Jing Chen, Kexin Ji, Yutong Cui, Weina Ge, Zhenyi Wang

**Affiliations:** ^1^ College of Life Sciences, North China University of Science and Technology, Tangshan, Hebei, China; ^2^ College of Management, North China University of Science and Technology, Tangshan, Hebei, China

**Keywords:** foxtail millet, NODULE-INCEPTION-like protein, transcription factor, structure, molecular evolution, codon bias

## Abstract

The NODULE-INCEPTION-like protein (NLP) family is a plant-specific transcription factor (TF) family involved in nitrate transport and assimilation in plants, which are essential for improving plant nitrogen use efficiency. Currently, the molecular nature and evolutionary trajectory of NLP genes in the C4 model crop foxtail millet are unknown. Therefore, we performed a comprehensive analysis of NLP and molecular evolution in foxtail millet by scanning the genomes of foxtail millet and representative species of the plant kingdom. We identified seven NLP genes in the foxtail millet genome, all of which are individually and separately distributed on different chromosomes. They were not structurally identical to each other and were mainly expressed on root tissues. We unearthed two key genes (*Si5G004100.1* and *Si6G248300.1*) with a variety of excellent characteristics. Regarding its molecular evolution, we found that NLP genes in Gramineae mainly underwent dispersed duplication, but maize NLP genes were mainly generated via WGD events. Other factors such as base mutations and natural selection have combined to promote the evolution of NLP genes. Intriguingly, the family in plants showed a gradual expansion during evolution with more duplications than losses, contrary to most gene families. In conclusion, this study advances the use of NLP genetic resources and the understanding of molecular evolution in cereals.

## 1 Introduction

Nitrogen is an important component of amino acids, nucleotides, chlorophyll, and hormones, and it is also an essential nutrient for all organisms (Tegeder and Masclaux-Daubresse, 2018; Li D. et al., 2024). The growth and development of crops depend on their ability to take up and utilize nitrogen. The main nitrogen source of terrestrial plants is nitrate. However, most terrestrial soils in the world contain less nitrogen, and the absorption and utilization of nitrogen by most terrestrial plants only account for 30%–50% of the nitrogen application amount (Chen et al., 2020). Therefore, to improve the utilization of nitrogen fertilizer (Liu et al., 2018), research on genes related to nitrogen uptake and transport has become increasingly popular in recent years. Plants and fungi are the only multicellular organisms capable of absorbing inorganic nitrogen. In higher plants, inorganic nitrogen is primarily absorbed from the soil in the form of NO^3-^ by various complex regulatory mechanisms evolved by plants (Crawford, 1995; Castaings et al., 2009). Among them, genes encoding nitrate transporter (NRT), nitrate reductase (NIA), and nitrite reductase (NIR) play an important role in the absorption and utilization of nitrate by plants (Forde, 2000; Li Y. et al., 2024). And the NODULE-INCEPTION-like protein (NLP) transcription factors play a central role in nitrate sensing and signaling. The NLP transcription factor family is a plant-specific transcription factor (TF) (Feng et al., 2020) family that participates in nitrate signal transduction and assimilation processes (Nishida and Suzaki, 2018; Gao et al., 2022).

The earliest research on NLP family can date back to the leguminous model plant *Lotus japonicus* nodule inception (NIN) (Schauser et al., 1999). The most obvious feature of NIN protein is a highly conserved long sequence composed of 60-amino acid, known as RWP-RK sequence (also called RWP × RK motif). Some highly homologous genes to NIN have been found in legumes, which were named NIN-like protein (NLP) (Borisov et al., 2003). NLP carries mainly the RWP-RK (Chardin et al., 2014) and PB1 (Sumimoto et al., 2007) conserved domains. NLP is homologous to NIN in the RWP-RK structural domain and the *N*-terminal region (Schauser et al., 2005; Suzuki et al., 2013). The RWP-RK structural domain is highly conserved, can bind and function with DNA, and its activity is independent of the nitrate signal (Chardin et al., 2014; Liu et al., 2018). The RWP-RK structure is an activation domain for transcription-mediated nitrate signaling, and the PB1 domain is located at the carboxyl terminus and can participate in protein-protein interactions (Ge et al., 2018). Moreover, the N-terminus of NLP has a highly conserved cGMP phosphodiesterase domain in addition to the RWP-RK domain, which may be involved in signal transduction or dimerization (Yu et al., 2023). There have been many genome-wide studies of the NLP gene family, such as *Arabidopsis thaliana* (Schauser et al., 2005), rice (*Oryza sativa*) (Schauser et al., 2005), pepper (Wu et al., 2023), alfalfa (Wu et al., 2023), cucumber seedlings (Li Y. et al., 2024), tea tree (Li D. et al., 2024), *Physcomitrella patens* (Crawford, 1995), wheat (Kumar et al., 2018), maize (*Zea mays*) (Ge et al., 2018), *Brassica napus* (Feng et al., 2020), Chinese cabbage (Chen et al., 2022a) and so on. Evolutionary studies of the Chinese cabbage NLP family have revealed that the origins of duplication of the NLP gene family in the genus Brassica were almost exclusively derived from the WGD type (Chen et al., 2022a), yet the molecular evolutionary characterization of the other plant taxa is not known.

Foxtail millet (*Setaria italica*) is an ancient diploid C4 gramineous model crop (Kumar et al., 2013) with a long history of cultivation. The process of humans cultivating wild weed green foxtail (*Setaria viridis* L.) into foxtail millet can be traced back to about 11,000 years ago (Li et al., 2022). Foxtail millet is an abiotic stress-tolerant plant with a short life cycle, which can be inbreeding and self-pollination. It can be grown as a food crop in the saline-prone regions of Asia and under adverse conditions such as drought and semi-drought conditions, as well as hay and fodder in Australia, southern Europe, South America, and North Africa (Sreenivasulu et al., 2004; Sharma and Niranjan, 2018). The genome of foxtail millet has been sequenced, and it has a small diploid genome (approximately 515 Mb) with a relatively small amount of repetitive DNA (Zhang G. et al., 2012; Bennetzen et al., 2012). Foxtail millet has gradually become a model species for studying gramineous crops (Doust et al., 2009; Yang et al., 2020) and provides data resources for studying the NLP gene family in foxtail millet.

In this study, we aimed to deepen our understanding of the functions of the NLP gene family in foxtail millet by conducting a genome-wide identification and bioinformatics analysis of the NLP gene family. The analysis included codon bias analysis, gene structure analysis, protein structure analysis, phylogenetic analysis, chromosome localization analysis, homology analysis, duplication type analysis, expansion analysis, and tissue expression analysis. The results of this analysis are expected to be fully applied and contribute to the research of the NLP gene family in foxtail millet.

## 2 Materials and methods

### 2.1 Data collection

We obtained the genome data for *A. thaliana* from the TAIR database (Berardini et al., 2015) (https://www.arabidopsis.org/), for rice from the Rice Genome Database (Ouyang et al., 2007) (http://rice.plantbiology.msu.edu/), and for *Chlorella variabilis* and *Chara braunii* from the NCBI database (https://www.ncbi.nlm.nih.gov/). Additionally, we downloaded genome data for *Chlamydomonas reinhardtii* (Merchant et al., 2007), *Volvox carteri* (Prochnik et al., 2010), *Ostreococcus lucimarinus* (Palenik et al., 2007), *Amborella trichopoda* (Project et al., 2013), *P. patens* (Lang et al., 2018), *Selaginella moellendorfii* (Banks et al., 2011), maize (Hirsch et al., 2016), sorghum (*Sorghum bicolor*) (McCormick et al., 2018) and foxtail millet (Bennetzen et al., 2012) in the JGI database (https://genome.jgi.doe.gov/).

After reviewing the literature (Jagadhesan et al., 2020), we identified six members of the NLP gene family in rice and obtained the nucleic acid and protein sequences for the rice NLP gene family. Using the six NLP sequences from rice as targets, we searched for candidate NLP gene family members in other species (*C*. *variabilis*, *C*. *braunii*, *C*. *reinhardtii*, *V*. *carteri*, *O*. *lucimarinus*, *P. patens*, *S*. *moellendorfii*, *A*. *trichopoda*, sorghum, maize, and *A. thaliana*) by performing a local tool Blastp (Altschul et al., 1990; Altschul et al., 1997) search with an e-value less than 1e^−5^ against all protein sequences in each species’ database. We then used the online tools Pfam (Finn et al., 2014) (http://pfam.xfam.org/) and SMART (Letunic et al., 2012) (http://smart.embl-heidelberg.de/) to confirm the presence of two conserved structural domains, RWP-RK and PB1. After filtering out the NLP sequences for each species, we manually modified the prefixes of the original IDs to the initials of the species’ Latin names to facilitate analysis.

### 2.2 Two-dimensional and three-dimensional structure of NLP family in foxtail millet

We utilized the online tool SOPMA (Geourjon and Deleage, 1995) (https://npsa-prabi.ibcp.fr/cgi-bin/npsa_automat.pl?page=npsa_sopma.html) to predict the two-dimensional structure of proteins. SOPMA’s basic principle for predicting amino acid secondary structure is based on the conserved and physicochemical properties of the sequence, which utilizes the self-similarity of the protein sequence. By comparing and analyzing the protein sequences, SOPMA can identify the repeats and conserved structures in the sequences and predict the secondary structure of the proteins. A significant improvement of the SOPMA method is that it takes into account sequence alignment information belonging to the same family, which makes the prediction results more accurate (Geourjon and Deleage, 1995). We uploaded the protein files of each of the seven foxtail millet NLP genes (Supplementary Material S1) to the SOPMA website with the default parameters (Number of conformational states: 4 (Helix, Sheet, Turn, Coil), Similarity threshold: 8, Window width: 17), and recorded the proportions of alpha helix, extended strand, beta turn, and randon coil in the results in tabular form.

PHYRE2 is a web-based tool that uses advanced remote homology detection methods to build 3D models of proteins, predict ligand binding sites, and analyze the effects of amino acid variants (e.g., nonsynonymous snps (nssnps) on the user’s protein sequence (Kelley et al., 2015). We used the online tool PHYRE2 (Kelley et al., 2015) (http://www.sbg.bio.ic.ac.uk/phyre2/html/page.cgi?id=index) to predict the three-dimensional structure of proteins. We separately uploaded the seven NLP protein sequences of foxtail millet to the PHYRE2 website with default parameters (Modelling Mode info icon: Normal, please tick as appropriate: Other), the final result was sent to the mailbox in the form of .pdb files, and the results were visualized using the native tool VMD (Humphrey et al., 1996) (version 1.9.4a51). We adjusted the color settings to helix-ColorID0, sheet-ColorID1, turn-ColorID4, and coil-ColorID7, with a transparent background color.

### 2.3 Promoter analysis of foxtail millet NLP genes

We extracted a 2000 bp sequence upstream of seven NLP genes from the foxtail millet genome file as promoter sequences using a self-design Python script ‘finally_promoter_genome.py’ (https://github.com/ChenHuilong1223/CFVisual/blob/main/finally_promoter_genome.py) based on the gff3 data of foxtail millet. The promoter sequences (Supplementary Material S2) were analyzed using the promoter analysis website PlantCARE (Lescot et al., 2002) (http://bioinformatics.psb.ugent.be/webtools/plantcare/html/), and the resulting promoter elements located on the negative strand were removed. The remaining promoter elements were classified by function according to the classification table. Using the Neighbor-joining method in MEGA6 (Tamura et al., 2013), seven NLP protein sequences of foxtail millet were compared, and the parameters were set as follows: repeat 1,000 times, Poisson model and pairwise deletion, with other parameters set to default. We exported the resulting tree file, which contained bootstrap values, and used CFVisual (Chen et al., 2022b) to visualize the results.

Subsequently, we used the online tool JASPAR (Castro-Mondragon et al., 2022) (https://jaspar.elixir.no/search?q=&collection=CORE&tax_group=plants) to cross-check the prediction of NLP transcription factor binding sites (TFBSs), thereby increasing the credibility of PlantCARE promoter analysis results. As far as we know, JASPAR is a comprehensive database website dedicated to predicting TFBSs, and its prediction results include experimental evidence and algorithm prediction. It is believed that the prediction results of *cis*-acting elements in the putative promoter sequence of foxtail millet NLP TFs include both TFBSs and non-TFBSs such as hormones. We chose the following scheme for cross-validation. We first investigated and classified all the *cis*-acting elements we identified, and then we selected the results of known TFBSs to verify in the JASPAR database. In the JASPAR CORE non redundant database from plants, we selected all the MYB TFs corresponding to the *cis*-acting element MBS, and predicted the binding sites with the seven NLP promoter sequences of foxtail millet, respectively. The relative contour score threshold was 100%. Finally, we removed the promoter located on the negative chain in the prediction results, and compared the prediction results with the PlantCARE promoter analysis results.

### 2.4 Tissue expression analysis of foxtail millet NLP genes

We downloaded the transcriptome data for the root, stem, leaf, and spica of Zhang Gu from the public database (Zhang G. et al., 2012) (https://www.ncbi.nlm.nih.gov/geo/query/acc.cgi?acc=GSE36391) and performed a two-way homology alignment of the foxtail millet NLP family sequences with the Zhang Gu sequences using the native tool Blastp. We then extracted the corresponding results with a self-made Perl script (Supplementary Program S1) and obtained the expression data (RPKM) and log2 (RPKM) for the NLP gene family in foxtail millet from the transcriptome data. Next, we used the online tool Venn (Chen et al., 2021) (http://www.ehbio.com/test/venn/#/) to perform statistical analysis on the NLP gene expression data of foxtail millet, and the online tool Hiplot (https://hiplot.com.cn/basic) to generate a tissue expression heat map for the foxtail millet NLP gene family. We also downloaded the transcriptome data for the rice expression matrix from the Rice Genome Database and calculated log2 (FPKM) values to generate a tissue expression heat map for the rice NLP gene family using the same methods.

### 2.5 Analysis of protein interactions in foxtail millet NLP proteins

We predicted the interaction of NLP family members with related proteins in foxtail millet by the online website STRING (Szklarczyk et al., 2021) (https://string-db.org/). We predicted interactions for each of the seven NLP families individually, with a minimum required interaction score set to high confidence (0.700) and the maximum number of interactors set to 5, with other parameters set to default values. Furthermore, we also uploaded all protein sequences of the NLP family in foxtail millet to the website in batches for prediction.

### 2.6 Analysis of GO and KEGG of NLP genes in foxtail millet

We performed gene ontology (GO) and Kyoto Encyclopedia of Genes and Genomes (KEGG) analyses on the seven NLP gene family members of foxtail millet using the agriGO v2.0 (Tian et al., 2017) (http://systemsbiology.cau.edu.cn/agriGOv2/) and KOBAS 3.0 (Bu et al., 2021) (http://kobas.cbi.pku.edu.cn/kobas3/?t=1) databases. We used Microsoft Office Excel to organize the GO function annotation results and KEGG analysis results.

### 2.7 Chromosome distribution analysis of the NLP family in foxtail millet and closely related crops

We counted the number of foxtail millet, rice, sorghum, and maize in each group based on the grouping of the phylogenetic tree. Then, we created a graph using Microsoft Excel with foxtail millet, rice, sorghum, and maize as the horizontal axis and the number as the vertical axis.

### 2.8 Duplication type analysis of NLP family in foxtail millet and closely related crops

We used the dupicate_gene_classifier program (Wang et al., 2012) in MCScanX to identify the five types of duplications (whole genome/segmental, tandem, proximal, dispersed, and singleton) of the whole genome and NLP gene families of foxtail millet, rice, sorghum, and maize. We also counted genome-wide collinear information tables with the NLP gene family using our previously published method (Chen et al., 2022a).

### 2.9 Codon bias analysis and ENC-Plot mapping of NLP families in foxtail millet and closely related crops

We downloaded the coding sequence (CDS) data of foxtail millet from the JGI database and extracted the CDSs of the NLP gene family in foxtail millet using a homemade Python program. We then screened the CDSs based on the following criteria: the sequence length was greater than or equal to 300 bp, the start codon was ATG (AUG), the stop codon was TAG (UAG), TAA (UAA), TGA (UGA), and the sequence did not contain other bases except A, T (U), G, C.

After the screening, we obtained the CDSs of the NLP genes in foxtail millet. We processed these seven CDSs with CodonW version 1.4.2 (https://sourceforge.net/projects/codonw/) and collated the results using Microsoft Office Excel. We used parameters such as codon adaptation index (CAI), effective number of codons (ENC), and relative synonymous codon usage (RSCU) for data processing. Using the *ggplot2* package in the R language to draw the ENC-plot diagram, the ENC value of each CDS is the ordinate, and the GC3 s is the abscissa to draw the two-dimensional scatter plot. The standard curve calculation formula was: ENC=2+GC3s+29/[GC3s2+(1—GC3s)2] (Wright, 1990). An RSCU value greater than 1 indicates that the codon is used more frequently, an RSCU equal to 1 indicates that the codon has no preference, and an RSCU less than 1 indicates that the codon is used less frequently (Sharp and Li, 1986). Similarly, we used the same method to analyze the codon bias of NLP genes in foxtail millet closely related species (rice, sorghum, and maize).

### 2.10 Determination of the optimal codon of the NLP family in foxtail millet and closely related crops

We used Microsoft Excel to determine the optimal codons. We sorted the genes based on their ENC preferences and selected 10% of genes at each end to determine the high and low expression genes. We then identified codons with a ∆RSCU (∆RSCU = high expression - low expression) greater than 0.08 in the high and low expression genes as high expression superior codons. Finally, we selected the codon which has the highest RSCU value (RSCU > 1) in each amino acid as the high frequency superior codon. If the codon was a high-expression superior codon, it was considered the optimal codon.

### 2.11 Construction of a phylogenetic tree of the NLP family of representative species in plants

We used the protein sequences of 50 NLP families in *C. braunii*, *O. lucimarinus*, *A. trichopoda*, *P. patens*, *S. moellendorfii*, *A. thaliana*, rice, maize, sorghum, and foxtail millet to construct a phylogenetic tree using the local tree-building software MEGA6. Using ClustalW in MEGA6 for multiple sequence alignment, default parameters. And the Maximum Likelihood tree construction method was selected. The parameters were set as follows: the best protein model was JTT + G + I with 1,000 repetitions and pairwise deletion. We then used the online tool EvolView (Zhang H. et al., 2012) (https://www.evolgenius.info/) to build a phylogenetic tree.

### 2.12 Analysis of selection pressure on NLP families of representative species in plants

We used a homemade Python program to delete the termination codons of the CDS files of the ten studied species and then compared them using MEGA6. We saved the comparison files to obtain the comparison files and then constructed a phylogenetic tree using the NJ method. We saved the tree file without Branch Length. Subsequently, positive selection analysis was performed by EasyCodeML (Gao et al., 2019). In the preset mode, the site model was used to select the positive selection site; in the custom mode, the free-ratio model of the branch model was made, and the branch with ω > 1 in the result was marked as the foreground branch, and the five foreground branches were a group. Then the free ratio model was changed to a double-ratio model. If the result was still ω > 1, the branch was positively selected.

### 2.13 Exon-intron analysis of NLP families of representative species in plants

We downloaded the GFF3 data for *A. thaliana* from the TAIR database, for rice from the Rice Genome Database, for *C. braunii* from the NCBI database, and for *O. lucimarinus*, *A. trichopoda*, *P. patens*, *S. moellendorfii*, maize, sorghum, and foxtail millet from the JGI database. We used a homemade Python script to extract the NLP gene structure information from the GFF3 data of each species and merged it into the same file. We then performed statistical analysis on the gene structure and visualized the results using CFVisual software.

### 2.14 Analysis of conserved motifs and structural domains of NLP families of representative species in plants

The MEME Suite web server (Bailey et al., 2009) (https://meme-suite.org/meme/tools/meme) can perform four types of motif analysis: motif discovery, motif-motif database searching, motif-sequence database searching and assignment of function. It is a good tool for discovering and searching sequence motifs. These sequence motifs represent features such as DNA binding sites and protein interaction domains. We uploaded 50 NLP protein sequences of the study species for motif discovery, and used the Multiple Em for Motif Elicitation (MEME) algorithm. We set the maximum value of the motif to 15 (Ge et al., 2018; Liu et al., 2018) and left the other parameters as default (Select the motif discovery mode: Classic mode, Select the sequence alphabet: DNA, RNA or Protein; Select the site distribution: Zero or One Occurrence Per Sequence (zoops)). We saved the MEME results (meme.xml) locally. Then we uploaded 50 NLP protein sequences of the study species to the Pfam database for analysis and saved the analysis results in text form. Finally, we used CFVisual software to draw the motif and domain together on a map based on the location information in the result files of MEME and Pfam. To determine the location of the motif and domain association.

### 2.15 Homology analysis of NLP families of representative species in plants

Homology analysis of the NLP family of ten species was performed using OrthoMCL (Li et al., 2003) software. The parameter settings were percent Match Cutoff of 75 and an e-value Exponent Cutoff of −10. We created orthologous network maps using the native software Cytoscape (Shannon et al., 2003), and homologous radar maps were created using Microsoft Excel.

### 2.16 Expansion analysis of the NLP family of representative species in plants

We identified the evolutionary relationships of the ten studied species containing NLP genes from the available literature (Geourjon and Deleage, 1995; Humphrey et al., 1996; Letunic et al., 2012; Kelley et al., 2015; Chen et al., 2021; Szklarczyk et al., 2021). We then constructed a species evolutionary tree using MEGA6 to understand the evolutionary process and genetic relationship among the studied species. Based on species and phylogenetic trees, we determined the gains and losses of NLP genes in the evolution of ten species through software Notung (Chen et al., 2000).

## 3 Results

### 3.1 Data collection

After a homology search, we identified seven sequences in the foxtail millet genome: *Si5G004100.1*, *Si3G084600.1*, *Si9G553000.1*, *Si8G074000.1*, *Si2G298700.1*, *Si1G094300.1*, and *Si6G248300.1*. We also identified nine sequences in the dicotyledonous plant *Arabidopsis* and nine, five, and seven sequences in the closely related species maize, sorghum, and rice, respectively. Three sequences were identified in the basal angiosperm *A. trichopoda*, while two and eight sequences were identified in the lower angiosperm *S. moellendorfii* and *P. patens*, respectively. Among the algae, we identified one sequence in each genome of *C. braunii* and *O. lucimarinus*. None were detected in *C. reinhardtii*, *C. variabilis* and *V. cariabilis*. By quantitatively comparing the results, we found a trend of NLP family size amplification from lower to higher plants. For instance, the number of NLPs in algae (*C. braunii*, *C. reinhardtii*, *C. variabilis*, *V. cariabilis*, and *O. lucimarinus*) ranged from zero to one, while lower plants (*S. moellendorfii*) had two NLPs, basal angiosperms (*A. trichopoda*) had three NLPs, and higher plants (*A. thaliana*, maize, sorghum, foxtail millet, and rice) had five to nine NLPs. Notably, the lower plant *P. patens* had more NLPs (eight), and maize had the most NLPs (nine) in the Graminaceae family.

### 3.2 Two-dimensional and three-dimensional structure of NLP family in foxtail millet

**FIGURE 1:**
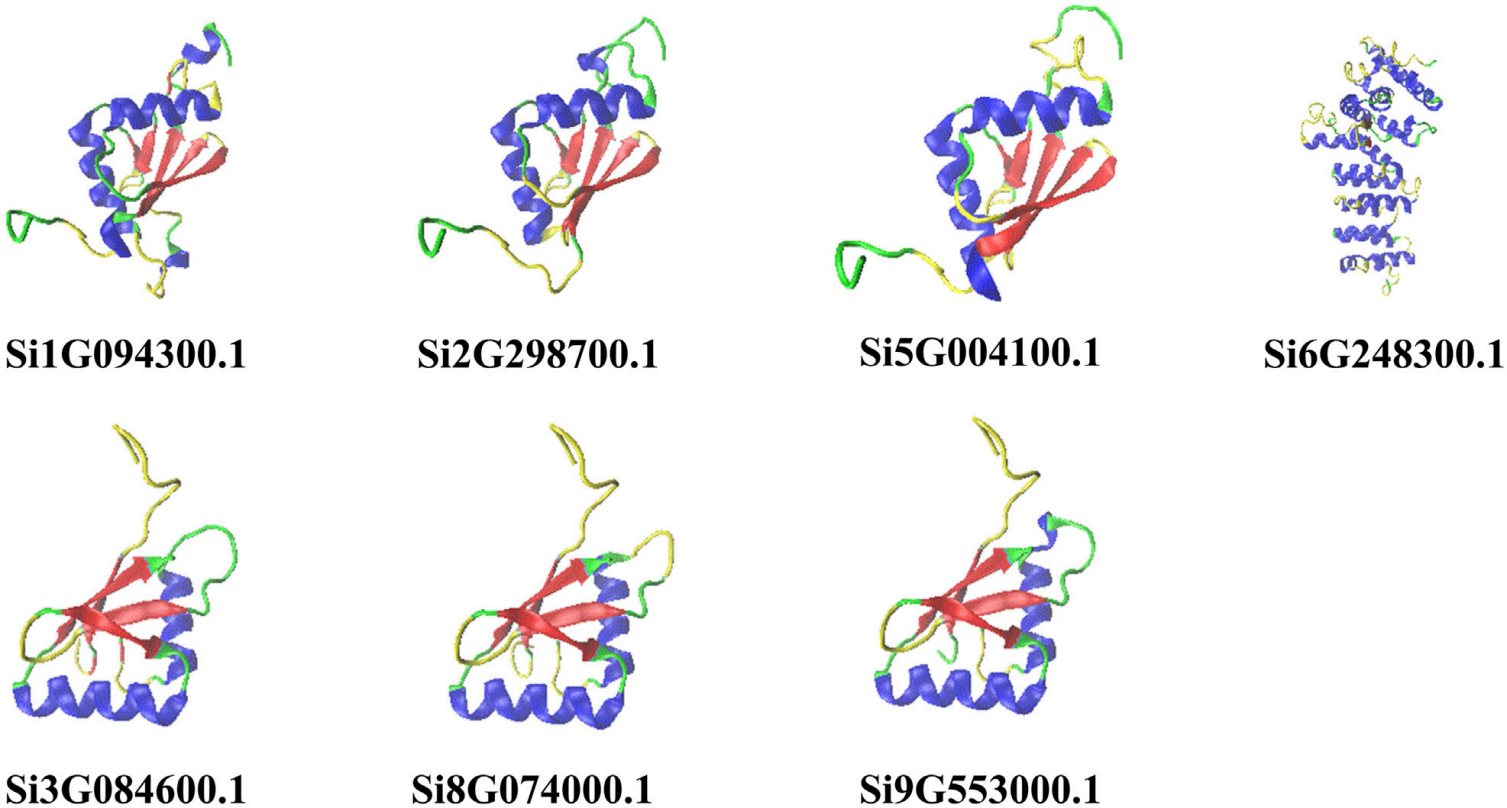
Prediction of the tertiary structure of NLP family proteins in foxtail millet. Note: Helix-blue; sheet-red; turn-yellow; coil-green.

We predicted the two-dimensional and three-dimensional structure of the NLP family in foxtail millet. According to the prediction analysis by the online tool SOPMA, all seven foxtail millet proteins had extremely similar percentages of two-dimensional structures (Table 1). Among them, α-helix and random coil were the dominant structures, with random coil accounting for the largest proportion (46.73%–55.61%), followed by α-helix (25.99%–34.18%), β-sheet (11.60%–14.67%), and β-turn being the smallest (4.15%–5.19%). To better understand the three-dimensional structure of the NLP proteins in foxtail millet, we used the online tool PHYRE2 to establish a three-dimensional protein model through homology modeling (Figure 1). We observed that the three-dimensional structures of the proteins encoded by genes *Si5G004100.1*, *Si2G298700.1*, and *Si1G094300.1* were similar. The three-dimensional structures of the proteins encoded by genes *Si3G084600.1*, *Si9G553000.1*, and *Si8G074000.1* were also similar. However, the three-dimensional structure of the protein encoded by gene *Si6G248300.1* differed from that of the remaining six protein sequences, with fewer β-folded parts.

**TABLE 1:** NLP family protein two-dimensional structure prediction results of foxtail millet.

Gene	Alpha helix (%)	Extended strand (%)	Beta turn (%)	Randon coil (%)
Si5G004100.1	25.99	13.69	4.71	55.61
Si3G084600.1	27.52	13.76	4.81	53.91
Si9G553000.1	31.11	12.55	4.15	52.18
Si8G074000.1	27.58	14.19	4.77	53.46
Si2G298700.1	29.35	14.67	5.19	50.79
Si1G094300.1	34.18	14.29	4.81	46.73
Si6G248300.1	31.43	11.60	5.14	51.82

### 3.3 Promoter analysis of foxtail millet NLP genes

**FIGURE 2:**
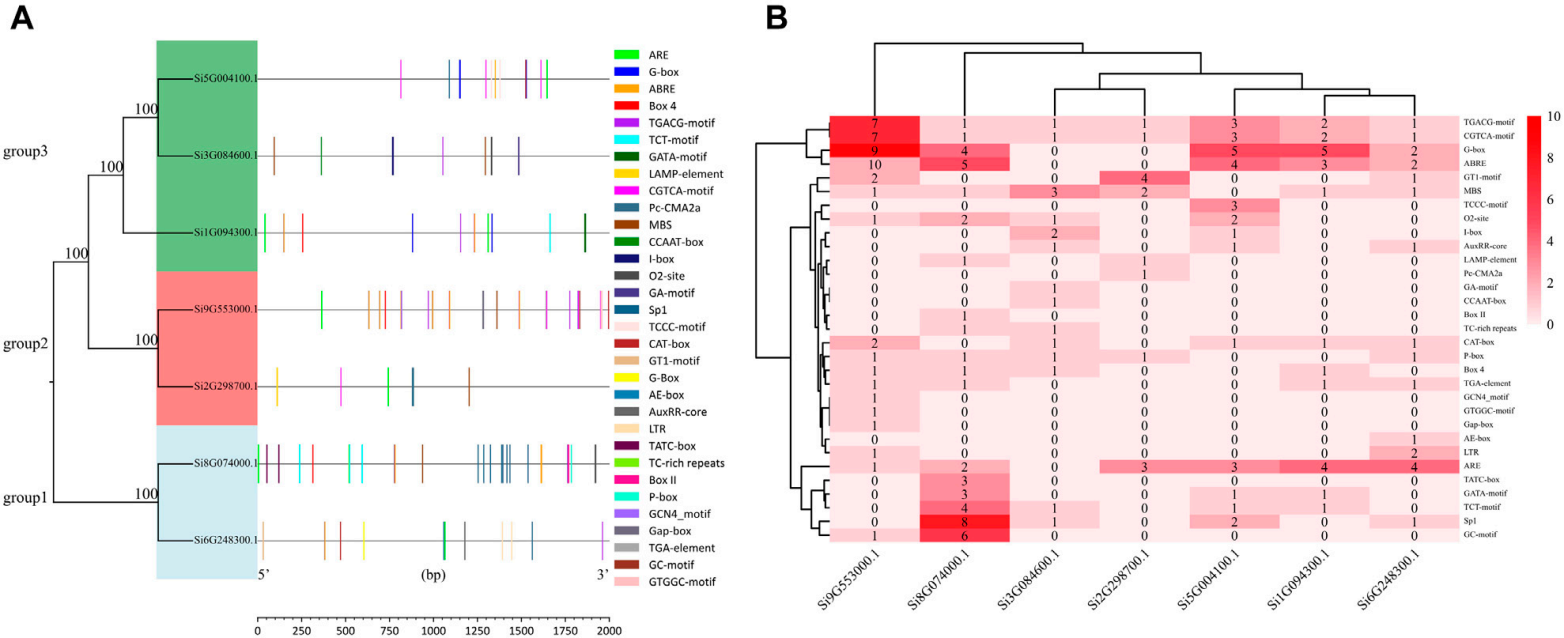
Analysis of promoter in foxtail millet NLP genes. **(A)** Distribution of promoter elements of the NLP gene family in foxtail millet. **(B)** Number of promoter elements of the NLP gene family in foxtail millet.

We selected the promoter sequences of seven NLP family genes and predicted the *cis-*acting elements of the 2 kb base sequence upstream of the transcription start site. The 31 *cis-*acting elements were divided into four major categories: light-responsive elements, stress-responsive elements, hormone-responsive elements, and other *cis-*acting elements (Figure 2A; Supplementary Table S1). We found a total of 196 *cis-*acting elements in the promoter sequences of the seven NLP families, including 15 light-responsive elements, six stress-responsive elements, seven hormone-responsive elements, and three other types of *cis-*acting elements. These include *cis-*acting regulatory elements associated with phloem expression, *cis-*acting regulatory elements involved in endosperm expression, and *cis-*acting regulatory elements involved in regulating maize alcohol-soluble protein metabolism. The promoter of *Si9G553000.1* was the only one containing all three *cis-*acting elements at the same time. Among the 15 light response elements, the G-box was the most abundant in the promoter of *Si9G553000.1*. Among the six stress response elements, the most abundant *cis-*acting element was ARE, which was present in all the promoters except for *Si3G084600.1*. Among the seven hormone-responsive action elements, ABRE, CGTCA-motif, and TGACG-motif were more abundant. As shown in Figure 2B, the promoter of *Si9G553000.1* contains many light-responsive action elements and hormone-responsive action elements. *Si8G074000.1* has more light-responsive action elements and stress-responsive action elements.

In the JASPAR prediction results, we found that the gene promoters (*Si3G084600.1*, *Si9G553000.1*, *Si8G074000.1*, *Si2G298700.1*, *Si1G094300.1*, and *Si6G248300.1*) all contained MBS elements with the numbers of 3, 16, 4, 1, 2, and 3, respectively. While gene *Si5G004100.1* did not contain MBS elements in its promoter. This prediction was highly consistent with the results of PlantCARE’s promoter analysis (Figure 2B). The reliability of PlantCARE’s promoter analysis results was also verified by the fact that all other six members of the foxtail millet NLP gene family have MBS elements, except for the absence of MBS elements in the promoter sequence of gene *Si5G004100.1* in PlantCARE’s analysis.

### 3.4 Tissue expression analysis of foxtail millet NLP genes

**FIGURE 3:**
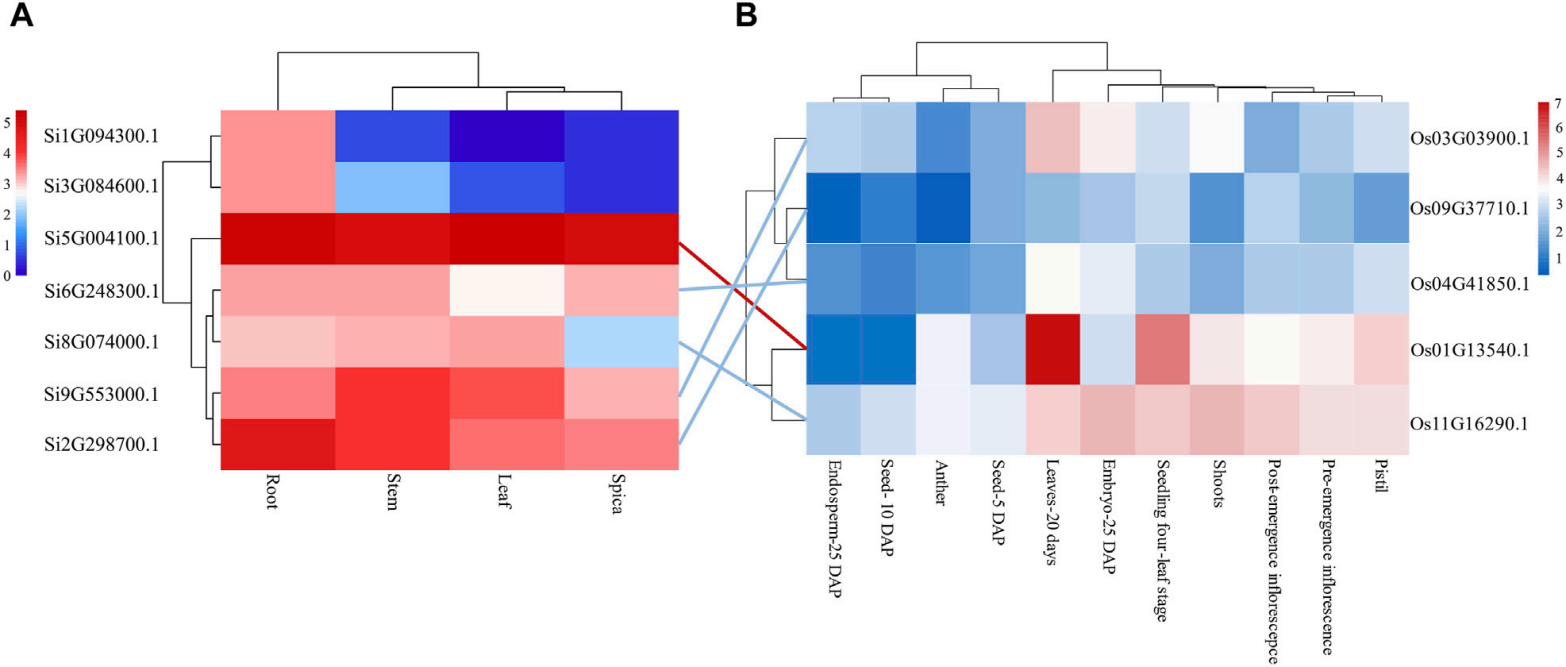
Tissue expression analysis of NLP gene family in foxtail millet and rice. **(A)** Tissue expression profile of foxtail millet NLP genes. **(B)** Tssue expression profile of rice NLP genes. Note: Lines represent orthologous gene pairs identified by OrthoMCL software. Red indicates that the expression level of the orthologous pair is higher, and blue indicates that the expression level of the orthologous pair is relatively low.

Analyzing gene expression helps to speculate about the function of genes. Therefore, we downloaded transcriptome data of the root, stem, leaf, and spica of foxtail millet from a public database and analyzed the tissue expression of the seven NLP genes in foxtail millet (Figure 3). The results showed that gene *Si1G094300.1* was expressed in three tissues (root, stem, and spica) but not leaf tissue. Except for gene *Si1G094300.1*, the remaining six NLP family members (*Si2G298700.1*, *Si3G084600.1*, *Si5G004100.1*, *Si6G248300.1*, *Si8G074000.1*, and *Si9G553000.1*) of foxtail millet were expressed to varying degrees in all four tissues (root, stem, leaf, and spica). Importantly, the gene *Si5G004100*.1 was highly expressed in all four tissues (root 36.30 RPKM, stem 28.65 RPKM, leaf 41.44 RPKM, spica 32.65 RPKM), indicating that the gene plays a great role in the growth and development of foxtail millet. The NLP gene family of foxtail millet exhibited tissue bias, mainly expressed in roots.

In addition, we analyzed the expression of NLP gene family in model organism rice. The NLP genes in rice were poorly expressed in Seed-5 DAP, Anther, Seed-10 DAP, and Endosperm-25 DAP but were expressed in all other tissues. However, the expression of genes *Os09G37710.1* and *Os04G41850.1* was low in each tissue. The gene *Os03G03900.1* was only weakly expressed in Leaves-20 days and Embryo-25 DAP. *Os01G13540.1* and *Os11G16290.1* were almost expressed in Pistil, Pre-emergence inflorescence, Post-emergence inflorescence, Shoots, Seedling four-leaf stage, Embryo-25 DAP, and Leaves-20 days. The gene *Os01G13540.1* had the highest expression in the leaves, no expression in the Post-emergence inflorescence, and lesser expression in the Embryo-25 DAP. There were five orthologous gene pairs in foxtail millet and rice, and the expression levels of the five orthologous gene pairs in leaves were similar. Among them, both the orthologous gene pairs *Si5G004100.1* and *Os01G13540.1* had higher expression in leaves, and both the orthologous gene pair *Si6G248300.1* and *Os04G41850.1* were not significantly expressed in leaves. However, the orthologous gene pairs *Si2G298700.1* and *Os09G37710.1* showed differential expression in leaf tissue, implying that the orthologous gene pairs underwent functional divergence after divergence between foxtail millet and rice.

### 3.5 Analysis of protein interactions in foxtail millet NLPs

**FIGURE 4:**
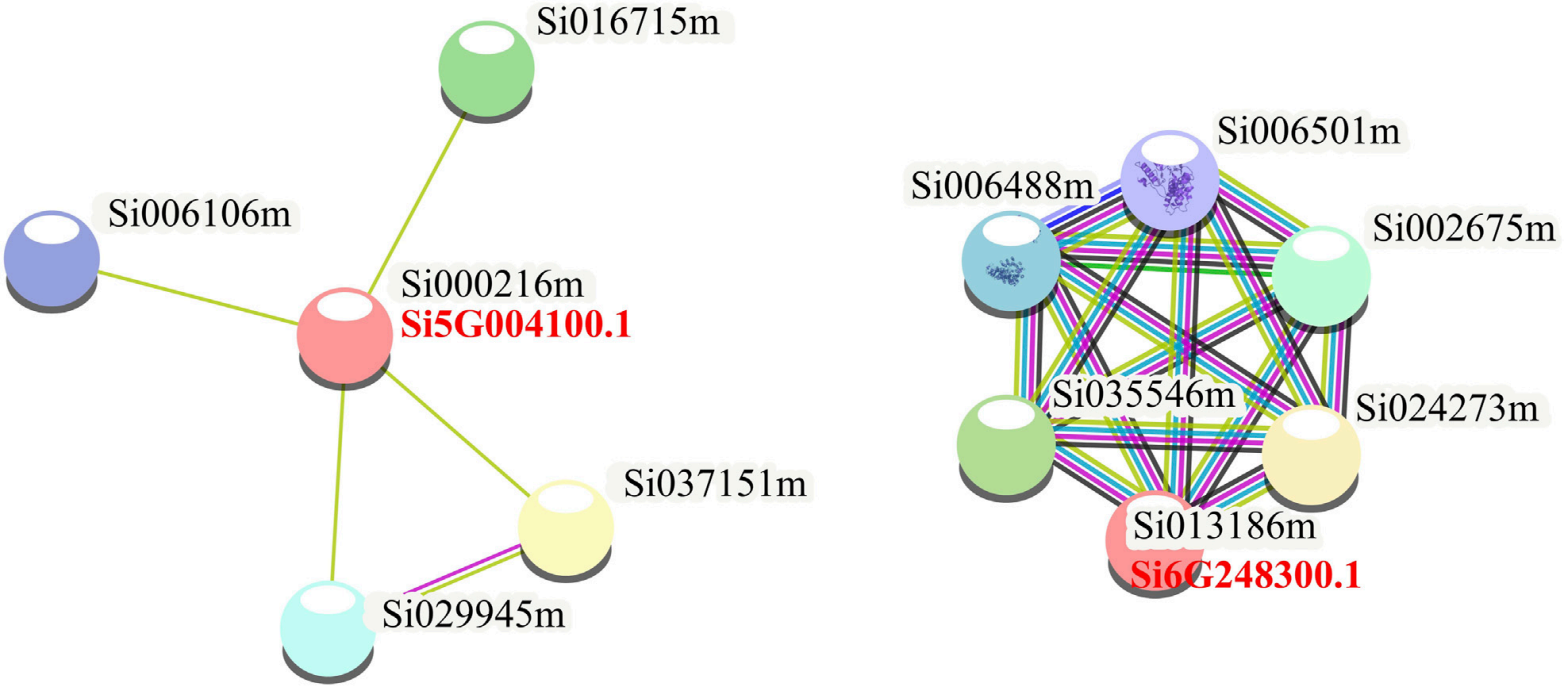
Protein interactions analysis of foxtail millet NLP gene family.

To further explore the function of NLP in foxtail millet, we performed protein-protein interaction analysis of the proteins expressed by the seven NLP genes of foxtail millet (Figure 4). The results showed that two NLP proteins (Si5G004100.1 and Si6G248300.1) could form protein-interacting networks with other proteins. Si5G004100.1 was able to form an interaction network with four proteins (Si016715m, Si037151m, Si029945m, and Si006106m). According to the annotation results of the String database, Si5G004100.1 interacted with iron redox protein nitrite reductase (Si016715m), SPX domain-containing protein/4 (Si037151m), HTH myb domain-containing protein (Si029945m), and foxtail millet nitrite transporter (Si006106m). Moreover, we found that Si6G248300.1 could form a protein complex interaction network with five proteins (Si035546m, Si006488m, Si006501m, Si002675m, and Si024273m). According to the annotation results of the String database, Si6G248300.1 interacted with proteasome subunit α (Si002675m), PCI domain-containing protein (Si035546m), two AAA domain-containing proteins (Si006488m and Si006501m), and MPN domain-containing protein (Si024273m). In addition, we also used the String database to construct a protein interaction network diagram of the seven NLP protein families as a whole and found that all members were independent of each other, indicating that the seven NLPs of foxtail millet may play independent biological roles in foxtail millet.

### 3.6 Analysis of GO and KEGG of NLP genes in foxtail millet

We also performed further functional predictions on the NLP family proteins of foxtail millet (Table 2). In the GO function annotation analysis results, the enriched GO entries in the NLP family of foxtail millet belong to molecular functions, and each GO entry contained seven NLP proteins, indicating that the foxtail millet NLP family played a role in binding activity or catalytic activity. In the KEGG function annotation analysis, only the Si6G248300.1 protein participated in the biological metabolism process.

**TABLE 2:** Analysis of GO and KEGG of NLP genes in foxtail millet.

GO ID and pathway ID	Term type	GO Term and pathway term	The number of genes	Corrected *p*-value	Gene ID
GO:0005515	MF	protein binding	7	0.0014	//Si6G248300//Si5G004100//Si2G298700//Si3G084600//Si8G074000//Si9G553000//Si1G094300
GO:0005488	MF	binding	7	0.29	//Si6G248300//Si5G004100//Si2G298700//Si3G084600//Si8G074000//Si9G553000//Si1G094300
sita03050		proteasome	1	0.014809	Si6G248300.1

### 3.7 Chromosome distribution analysis of the NLP family in foxtail millet and closely related crops

**FIGURE 5:**
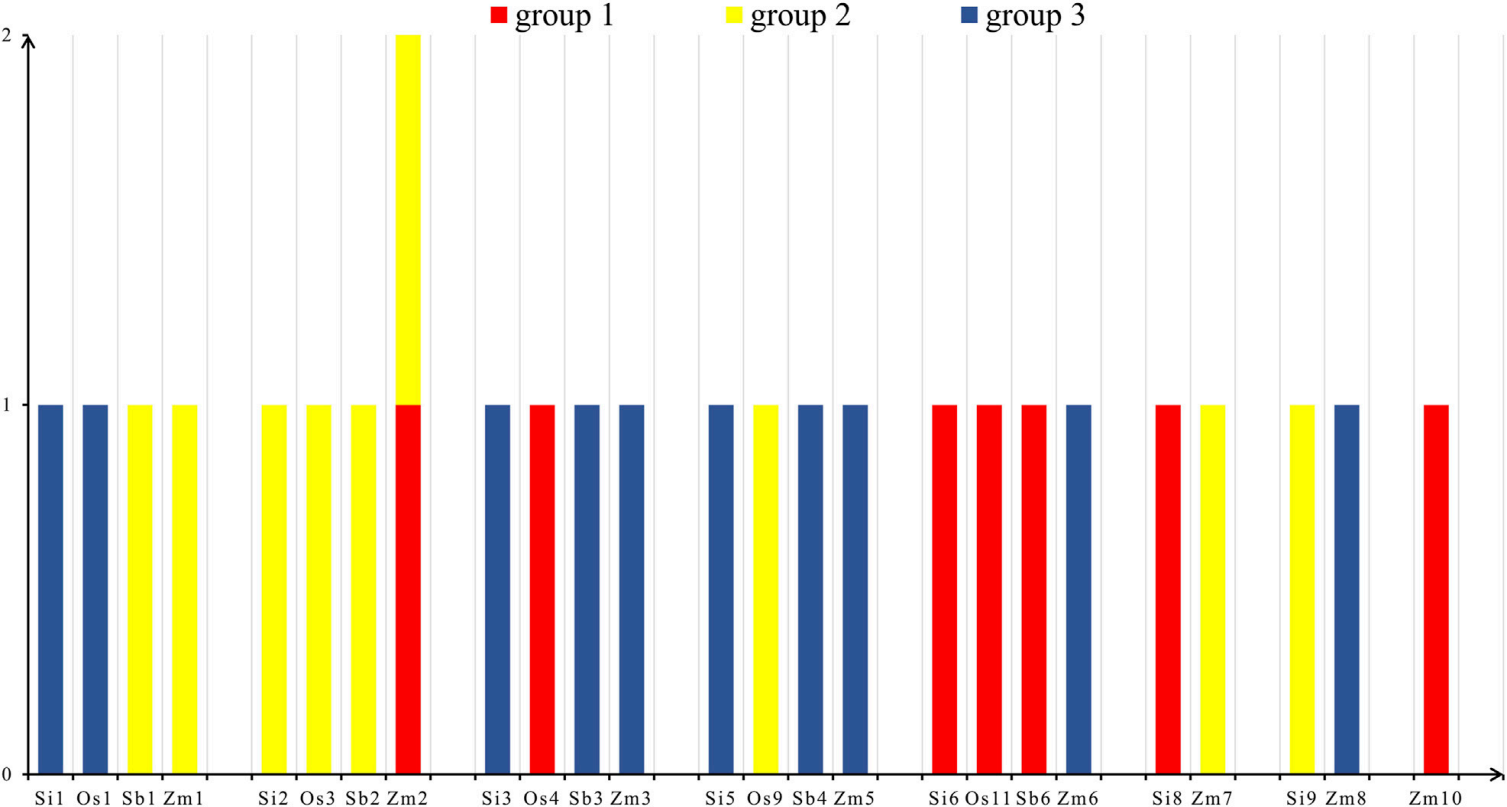
Chromosome distribution. Note: The horizontal axis is the four species of foxtail millet, rice, sorghum and maize, and the vertical axis is the number of NLP genes. Os, Si, Sb and Zm are abbreviations of rice, foxtail millet, sorghum and maize, respectively.

According to the statistical results (Figure 5), we found that there were seven NLP genes in group 1, nine NLP genes in group 2, and ten NLP genes in group 3. The seven NLP genes of foxtail millet were located on chromosomes 1 (*Si1G094300.1*), 2 (*Si2G298700.1*), 3 (*Si3G084600.1*), 5 (*Si5G004100.1*), 6 (*Si6G248300.1*), 8 (*Si8G074000.1*), and 9 (*Si9G553000.1*). Meanwhile, the five NLP genes of rice are distributed on five chromosomes: chromosomes 1, 3, 4, 9, and 11, respectively. The five NLP genes of sorghum were distributed on chromosomes 1, 2, 3, 4, and 6. The nine genes of maize were distributed on chromosomes 1, 2, 3, 5, 6, 7, 8, and 10, respectively, with two NLP genes on chromosome 2. On the chromosomes of the four species, the distribution of NLP genes is relatively dispersed and there is no clustering phenomenon.

### 3.8 Duplication type analysis of NLP family in foxtail millet and closely related crops

We investigated the duplication types of NLP genes in four species, including rice, foxtail millet, sorghum, and maize (Table 3). We found that none of the species had singleton, proximal, or tandem duplication types for NLP genes. Dispersed duplication was the main type of NLP gene duplication in rice and sorghum. In foxtail millet, five genes underwent dispersed duplication, while two genes experienced WGD or segmental duplication. Nine NLP genes were identified in maize, of which three genes underwent dispersed duplication, and six genes experienced WGD or segmental duplication. In the collinearity analysis of the four species (Table 4), we found that the proportion of NLP genes in collinear blocks and in genome-wide collinear blocks was different. The NLP genes of rice and sorghum were not in collinear blocks, and the proportion of collinear blocks in NLP genes of foxtail millet and maize (28.57% and 66.67%) was higher than that in genome (17.23% and 19.25%). These results provide insight into the evolution of NLP genes.

**TABLE 3:** Analysis of duplication types of NLP families.

Species	Singleton		Dispersed		Proximal		Tandem		WGD or segmental		Total	
	Genome	NLP	Genome	NLP	Genome	NLP	Genome	NLP	Genome	NLP	Genome	NLP
*Os*	8,758	0	33,184	5	3,965	0	3,822	0	6,072	0	55,801	5
*Si*	5,541	0	15,806	5	2,223	0	4,585	0	6,109	2	34,264	7
*Sb*	7,384	0	16,178	5	2,233	0	4,165	0	5,504	0	35,464	5
*Zm*	13,597	0	29,377	3	2,656	0	2,458	0	11,871	6	59,959	9

**TABLE 4:** Whole gene and NLP collinear block analysis.

Species	All genes				NLP genes			
	Total collinear blocks	Gene number in collinear blocks	Total genes	Percentage (%)	Collinear blocks contained NLP gene	NLP genes in collinear block	Total NLP genes	Percentage (%)
*Os*	180	5,731	55,801	10.27	0	0	5	0.00
*Si*	177	5,905	34,264	17.23	1	2	7	28.57
*Sb*	136	5,161	35,464	14.55	0	0	5	0.00
*Zm*	394	11,540	59,959	19.25	3	6	9	66.67

### 3.9 Codon bias analysis and ENC-Plot mapping of NLP families in foxtail millet and closely related crops

Studying the codon bias of gene families can provide valuable references for transgenic technology and also offer a new perspective for understanding the evolutionary history of a family. Maize, sorghum, and rice are closely related to foxtail millet and are common economic crops. To deepen our understanding of the evolution of these four species, we analyzed the codon bias of the NLP gene family in foxtail millet and its related species. We followed the literature standard (Wu et al., 2007) to reduce calculation errors and screened seven suitable CDSs of the NLP family of foxtail millet (Supplementary Table S2). Using CodonW, we obtained the results shown in Table 5, which includes the ENC used in the gene. The reference range of ENC value is 20–61, which can reflect the degree of preference for the unbalanced use of synonymous codons. The lower the ENC value, the stronger the preference. The ENC values of the NLP family genes of foxtail millet, rice, sorghum, and maize ranged from 45.55 to 58.74, 54.32 to 58.58, 40.74 to 58.6, and 41.64 to 59.39, respectively, with mean values of 55.3071, 56.512, 52.804, and 55.2756, respectively. The ENC value range and the average value of the four species were close to 60, indicating weak codon bias in the NLP family genes of these species.

**TABLE 5:** Codon bias parameters.

Species	Statistic	T3s	C3s	A3s	G3s	CAI	CBI	Fop	ENC	GC3s	GC
*Si*	range	0.1695–0.4161	0.2323–0.4757	0.131–0.3268	0.2564–0.4344	0.205–0.258	−0.044–0.157	0.397–0.512	45.55–58.74	0.4–0.748	0.451–0.64
	average	0.3282	0.3229	0.2772	0.3163	0.225	0.0269	0.4377	55.3071	0.508	0.5057
*O*s	range	0.2941–0.4155	0.247–0.3347	0.2452–0.339	0.2485–0.3652	0.199–0.233	−0.005∼-0.01	0.382–0.451	54.32–58.58	0.394–0.55	0.489–0.462
	average	0.3539	0.2818	0.3122	0.3017	0.211	−0.0182	0.4124	56.512	0.4598	0.482
*Sb*	range	0.1278–0.3947	0.2837–0.5192	0.1045–0.348	0.2609–0.4566	0.211–0.268	−0.043–0.197	0.397–0.535	40.74–58.6	0.432–0.805	0.469–0.662
	average	0.2975	0.3564	0.2644	0.325	0.2372	0.0612	0.4584	52.804	0.5442	0.5228
*Zm*	range	0.1297–0.4194	0.2411–0.4933	0.0978–0.3599	0.2437–0.4711	0.2–0.25	−0.042–0.193	0.397–0.53	41.64–59.39	0.373–0.806	0.433–0.676
	average	0.3129	0.3358	0.2737	0.3171	0.2204	0.033	0.4403	55.2756	0.5219	0.5122

Note: Fop, the optimal codon usage frequency.

The CAI measures the codon preference of a gene concerning a group of highly expressed genes. CAI values range from 0 to 1, with values closer to 1 indicating that the gene uses codons that are exclusively preferred by highly expressed genes. The CAI values of the NLP family genes in foxtail millet ranged from 0.205 to 0.258, with an average of 0.225. For rice, the CAI values of NLP family genes ranged from 0.199 to 0.233, with an average of 0.211. In sorghum, the CAI values of NLP family genes ranged from 0.211 to 0.268, with an average of 0.2372. In maize, the CAI values of NLP family genes ranged from 0.2 to 0.25, with an average of 0.2204. The codon bias index (CBI) is used to elucidate the components of all optimal codons in a particular gene. The CBI values of NLP family genes in foxtail millet ranged from −0.044 to 0.157, with an average of 0.0269. For rice, the CBI values of NLP family genes ranged from −0.005 to −0.01, with an average of −0.0182. In sorghum, the CBI values of NLP family genes ranged from −0.043 to 0.197, with an average of 0.0612. In maize, the CBI values of NLP family genes ranged from −0.042 to 0.193, with an average of 0.033. GC content of the third base of codon (GC3s) is another measure of codon preference. In monocots and dicots, the smaller the GC3s, the greater the influence of natural selection on codon preference (Gu et al., 2004).

Table 6 lists four species, each with a range of frequency (T3s) for the third base T of their synonymous codon: 0.1695–0.4161, 0.2941–0.4155, 0.1278–0.3947, and 0.1297–0.4194, respectively. The third base A also has a frequency range (A3s) in each species: 0.131–0.3407, 0.2452–0.339, 0.1045–0.348, and 0.0978–0.3599, respectively. Additionally, the third base G (G3s) has a frequency range of 0.2564–0.4344, 0.2485–0.3652, 0.2609–0.4566, and 0.2437–0.4711, respectively, while the frequency range of the third base C (C3s) is 0.2323–0.4757, 0.247–0.3347, 0.2837–0.5192, and 0.2411–0.4933, respectively. The frequency range of the third base GC (GC3s) for the four species is as follows: 0.4–0.748, 0.394–0.55, 0.432–0.805, and 0.373–0.806, respectively. The frequency range of the total codon GC (GC) is 0.451–0.64, 0.489–0.462, 0.469–0.662, and 0.433–0.676, respectively. Overall, there appears to be no clear base preference in the coding region of the NLP family genes of these four species, nor in the selection of bases in the third position of their codons.

**TABLE 6:** Exon—intron structure information of NLP family.

Group	Gene	Length	Intron	CDS	UTR	Group	Gene	Length	Intron	CDS	UTR
group 3	*Sb03G003700.1*	12,323	4	5	1	group 2	*Zm2G048582_P01*	5,233	3	4	2
	*Zm2G375675_P01*	6,192	4	5	2		*Sb02G302500.1*	5,350	5	4	4
	*Zm2G475305_P01*	4,391	4	5	2		*Zm2G053298_P01*	5,333	4	4	2
	*Si5G004100.1*	5,891	4	5		group 1	*At3G59580.1*	3,716	5	5	3
	*Os01G13540.1*	5,582	4	5	2		*At2G43500.1*	4,715	7	6	4
	*Si3G084600.1*	6,431	4	5	2		*Amscaffold00066.150*	8,425	4	5	0
	*Zm2G176655_P01*	9,204	4	5	2		*Os11G16290.1*	6,997	4	5	1
	*Amscaffold00058.115*	6,863	3	4	0		*Si8G074000.1*	4,668	5	5	3
	*At1G64530.1*	3,421	5	6	2		*Os04G41850.1*	6,468	5	5	3
	*At4G24020.1*	4,242	4	5	2		*Si6G248300.1*	5,424	9	10	2
	*Amscaffold00080.66*	8,312	3	4	0		*Zm2G031398_P02*	8,724	9	10	0
	*Si1G094300.1*	3,192	4	5	2		*Sb06G148100.1*	5,881	4	5	2
	*Sb04G038000.1*	3,520	4	5	2		*Zm2G105004_P01*	5,045	4	5	2
	*Zm2G042278_P01*	3,286	3	4	2		*Sm172537*	3,057	3	4	1
group 2	*At2G17150.1*	4,418	4	4	3		*Cb84175.1*	17,691	1	2	2
	*At4G35270.1*	3,487	3	4	1		*Pp3c17_4370*	6,312	2	3	2
	*At4G38340.1*	3,119	3	4	0		*Pp3c17_4375*	6,068	3	3	3
	*At1G76350.1*	3,510	4	4	3		*Pp3c12_2070*	6,281	2	3	2
	*At1G20640.1*	3,659	4	4	3		*Pp3c9_14600*	6,907	4	4	3
	*Sb01G552900.1*	4,488	4	4	2		*Pp3c15_9180*	6,254	4	4	3
	*Zm2G109509_P01*	4,700	5	5	1		*Pp3c19_2670*	7,268	3	4	2
	*Si9G553000.1*	5,643	5	4	4		*Pp3c22_6370*	6,052	3	4	2
	*Os03G03900.1*	4,629	4	4	3		*Pp3c22_6360*	5,840	3	4	2
	*Os09G37710.1*	5,194	4	4	3		*Sm61084*	2019	3	4	0
	*Si2G298700.1*	5,211	4	5	2		*Ol24740*	2,172	0	1	0

**FIGURE 6:**
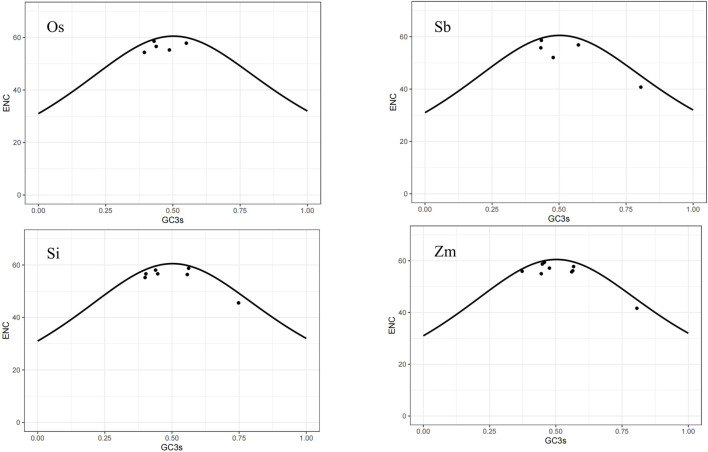
ENC-plot diagram.

The ENC-plot is a useful tool for detecting the impact of base composition on codon bias. A gene distributed along or near the standard curve suggests that the codon bias of the gene is solely influenced by mutations. Conversely, if a gene falls far from the standard curve, it indicates that the codon bias of the gene is affected by selection pressure and other factors. Based on the ENC-plot diagram for the four species (Figure 6), it can be observed that most of their genes are located close to or below the standard curve. We can draw the following inference: compared with base mutations, natural selection and other factors had a more significant effect on the codon bias of the NLP gene family.

### 3.10 Determination of the optimal codon of the NLP family in foxtail millet and closely related crops

RSCU is the ratio of the observed value of synonymous codons to the average expected value of synonymous codon usage in a sample, which intuitively reflects the degree of codon usage bias independent of amino acid usage and codon abundance (Sharp and Li, 1986). We selected one gene from each end (10%, rounded) and sorted it according to the ENC value from small to large, so as to obtain the genes with high expression and low expression in the NLP family of each species. The highly expressed gene of foxtail millet was *Si1G094300.1*, and the weakly expressed gene was *Si9G553000.1*. Similarly, the highly expressed gene of rice was *Os04G41850.1*, and the weakly expressed gene was *Os01G13540.1*. For sorghum, the highly expressed gene was *Sb04G038000.1*, and the weakly expressed gene was *Sb03G003700.1*. Lastly, the highly expressed gene of maize was *Zm2G042278_P01*, and the weakly expressed gene was *Zm2G475305_P01*. Preference libraries were established separately by species, and highly expressed superior codons were obtained for each species based on ΔRSCU >0.08 (27 for foxtail millet, 22 for rice, 25 for sorghum, and 28 for maize). Next, optimal codons were identified for each species based on the highest RSCU values of codons in each amino acid (Supplementary Table S3).

**FIGURE 7:**
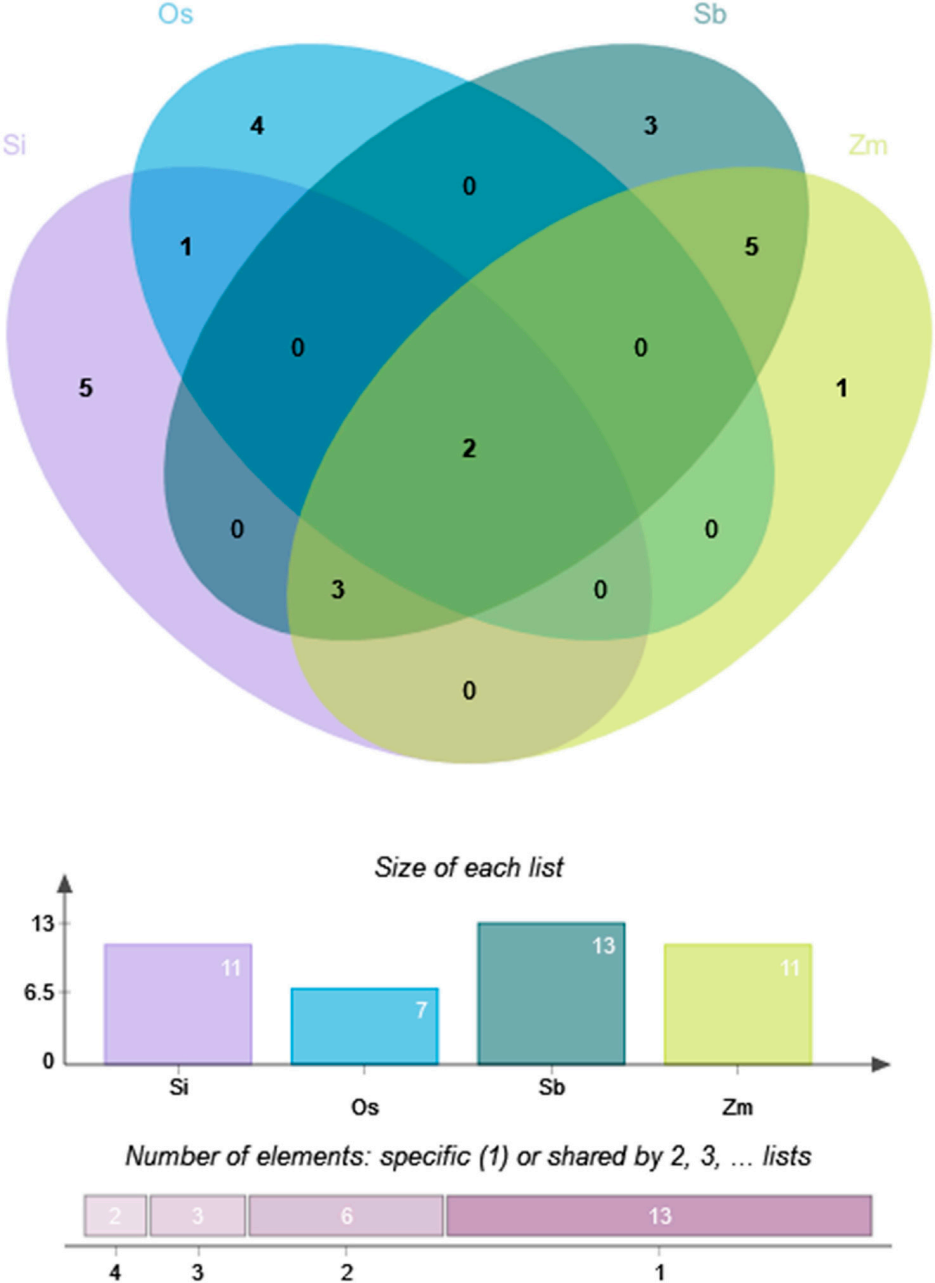
Optimal codon Venn diagram of NLP family in foxtail millet and closely related crops. Note: Os, Si, Sb and Zm are abbreviations of rice, foxtail millet, sorghum and maize, respectively.

Foxtail millet had 11 optimal codons, with six ending in A/U (T) and five ending in G/C; rice had seven optimal codons, with four ending in A/U (T) and three ending in G/C; sorghum had 13 optimal codons, all ending in G/C; maize had 11 optimal codons, all ending in G/C. Therefore, compared to sorghum and maize, the optimal codon bias of foxtail millet and rice was weaker. Figure 7 shows that the four species shared two optimal codons (AAG and UUC). In addition, foxtail millet, sorghum, and maize shared three optimal codons (CUG, UAC, and CAG). Sorghum and maize had five identical optimal codons (AUC, AAC, GAG, UGC, and GGC). Foxtail millet and rice shared one identical optimal codons (CCA).

### 3.11 Phylogenetic tree construction and selection pressure analysis of NLP family of representative species in plants

**FIGURE 8:**
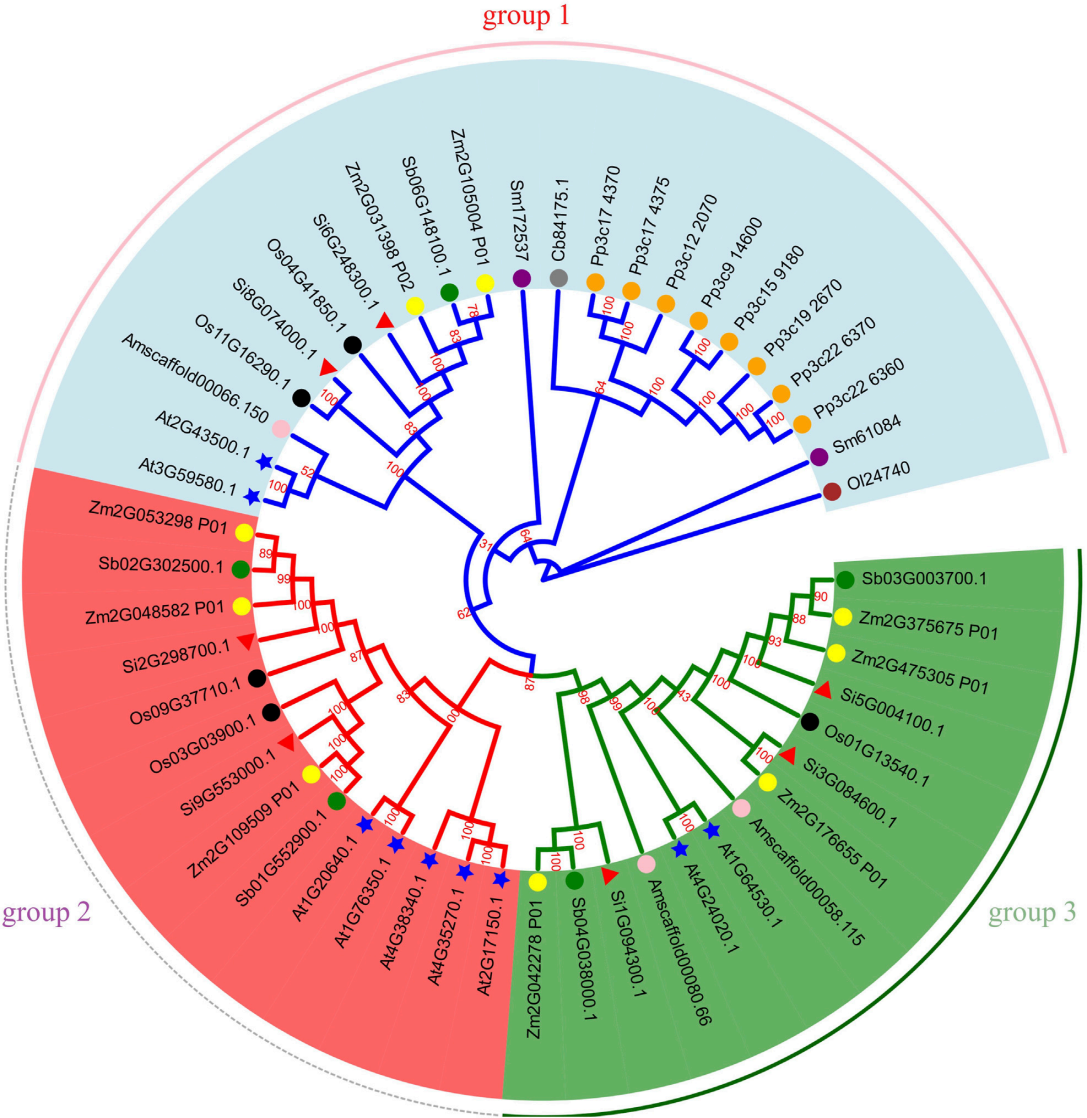
Phylogenetic tree of NLP gene family. Note: Gene IDs show their respective sources: Os represents rice, Si represents foxtail millet, Sb represents sorghum, Zm represents maize, Am represents *A. trichopoda*, At represents *A. thaliana*, Sm represents *S. moellendorfii*, Cb represents *C. braunii*, Pp represents *P. patens*, Ol represents *O. lucimarinus*. We use shape and color to distinguish different species. Green circles, yellow circles, black circles, pink circles, blue stars, orange circles, purple circles, brown circles and red triangles represent sorghum, maize, rice, *A. trichopoda*, *A. thaliana*, *P. patens*, *S. moellendorfii*, *O. lucimarinus* and foxtail millet, respectively. The number on the branch is the bootstrap value.

To explore the phylogenetic relationship of NLP, we aligned all NLP protein sequences of ten species (*A. thaliana*, maize, sorghum, foxtail millet, rice, *A. trichopoda*, *S. moellendorfii*, *P. patens*, *C. braunii*, and *O. lucimarinus*) containing NLP and constructed a phylogenetic tree (Figure 8). Based on previous classification criteria (Schauser et al., 2005; Hachiya et al., 2011) and the topology of the phylogenetic tree, we divided it into three groups. Group 1 contained two foxtail millet NLP genes (*Si6G248300.1* and *Si8G074000.1*), two *A. thaliana* NLP genes, one sorghum NLP gene, two maize NLP genes, two rice NLP genes, two *S. moellendorfii* NLP genes, eight *P. patens* NLP genes, one *A. trichopoda* NLP gene, one *C. braunii* NLP gene, and one *O. lucimarinus* NLP gene. Group 2 contained two foxtail millet NLP genes (*Si2G298700.1* and *Si9G553000.1*), three maize NLP genes, two sorghum NLP genes, two rice NLP genes, and five *A. thaliana* NLP genes. Group 3 contained three foxtail millet NLP genes (*Si1G094300.1*, *Si3G084600.1*, and *Si5G004100.1*), four maize NLP genes, two sorghum NLP genes, one rice NLP gene, two *A. trichopoda* NLP genes, and two *A. thaliana* NLP genes.

Based on the phylogenetic tree and sequence alignment files, we analyzed the selection pressure of representative plant species in the NLP family. The results showed that under the site model condition, no amino acid sites under positive selection pressure were detected (Supplementary Table S4). In the branch model, we labeled four foreground branches for the double-ratio model based on the results of the free-ratio model. However, the results did not identify any branches that were subjected to positive selection pressure.

### 3.12 Exon-intron analysis of NLP families of representative species in plants

We analyzed the differences in gene structure among ten species and used CFVisual software to determine the number of NLP gene structural features for each species (Table 6; Supplementary Figure S1). Among the five NLP sequences of foxtail millet, all except for *Si8G074000.1* and *Si9G553000.1* had one untranslated region (UTR) sequence at both ends. In group 1, *Si6G248300.1* had ten CDS and nine intron sequences, while *Si8G074000.1* had five CDS and five intron sequences. In group 2, *Si2G298700.1* had five CDS and four intron sequences, while *Si9G553000.1* had four CDS and five intron sequences. The three NLP sequences in group 3 contained five CDS and four intron sequences.

Among the nine NLP sequences in *A. thaliana*, *At4G38340.1* lacked a UTR sequence, *At4G35270.1* had one UTR sequence, *At1G64530.1* and *At4G24020.1* both had two UTR sequences, *At2G43500.1* had four UTR sequences, and the remaining four NLP sequences had three UTR sequences. In group 1, *At3G59580.1* had five CDS and five intron sequences, while *At2G43500.1* had six CDS and seven intron sequences. In group 2, *At4G35270.1* and *At4G38340.1* had four CDS and three intron sequences, while *At2G17150.1*, *At1G76350.1*, and *At1G20640.1* had four CDS and four intron sequences. In group 3, *At1G64530.1* had six CDS and five intron sequences, and *At4G24020.1* had five CDS and four intron sequences.

Among the nine NLP sequences of maize, *Zm2G031398_P02* lacked a UTR sequence, and *Zm2G109509_P01* had one UTR sequence, while the remaining seven NLP sequences had two UTR sequences. In group 1, *Zm2G105004_P01* had five CDS and four intron sequences, and *Zm2G031398_P02* had one CDS and nine intron sequences. In group 2, *Zm2G109509_P01* had five CDS and five intron sequences, while *Zm2G048582_P01* and *Zm2G053298_P01* had four CDS and three or four intron sequences. In group 3, except for *Zm2G042278_P01*, which had four CDS and three intron sequences, the remaining three NLP sequences had five CDS and four intron sequences.

Among the five NLP sequences of sorghum, *Sb03G003700.1* had one UTR sequence, and *Sb02G302500.1* had four UTR sequences, while the other three NLP sequences had two UTR sequences. In group 1, *Sb06G148100.1* had five CDS and four intron sequences; *Sb02G302500.1* had four CDS and five intron sequences, and *Sb01G552900.1* had four CDS and four intron sequences. In group 3, both *Sb03G003700.1* and *Sb04G038000.1* had five CDS and four intron sequences.

Among the five NLP sequences in rice, *Os11G16290.1* had one UTR sequence, and *Os01G13540.1* had two UTR sequences, while the remaining three NLP sequences had three UTR sequences. In group 1, *Os04G41850.1* had five CDS and five intron sequences, and *Os11G16290.1* had five CDS and four intron sequences. In group 2, *Os03G03900.1* and *Os09G37710.1* had four CDS and four intron sequences. In group 3, *Os01G13540.1* had five CDS and four intron sequences.

The three NLP sequences of *A. trichopoda* lacked a UTR sequence. In group 1, *Amscaffold00066.150* had five CDS and four intron sequences, while in group 3, *Amscaffold00080.66* and *Amscaffold00058.115* had four CDS and three intron sequences.

Both of the NLP sequences in *S. moellendorfii*, *Sm61084* and *Sm172537*, belong to group 1. *Sm61084* lacked a UTR sequence, while *Sm172537* had only one UTR sequence. Both sequences contained four CDS and three intron sequences.

Among the eight NLP sequences of *P. patens*, *Pp3c17_4375*, *Pp3c9_14600*, and *Pp3c15_9180* had three UTR sequences, while the remaining five NLP sequences had two UTR sequences. All eight NLP sequences were in group 1. *Pp3c17_4375* had three CDS and three intron sequences, *Pp3c17_4370* and *Pp3c12_2070* had three CDS and two intron sequences. *Pp3c9_14600* and *Pp3c15_9180* had four CDS and four intron sequences, and the other three NLP sequences had four CDS and three intron sequences.

*C. braunii* had only one NLP sequence, namely, *Cb84175.1*, which had two UTR sequences. It was located in group 1 and had two CDS and one intron sequence. *O. lucimarinus* had one NLP sequence, *Ol24740*, which was located in group 1 and had only one CDS with no UTR or intron sequences.

Among the researched species, most NLP gene families had a UTR sequence at each end of the sequence, and most gene families had one more intron sequence than CDS. The UTR at the 5′end of *Sb06G148100.1* was the longest (1,149 bp), while *At2G43500.1* had the shortest UTR (21 bp). The UTR at the 3′end of *Pp3c19_2670* was the longest (1972 bp), and *At3G59580.1* had the shortest UTR (45 bp). The CDS of the *Cb84175.1* gene of *C. braunii* was the longest (3,285 bp), and the intron inserted into the *Cb84175.1* sequence was the longest (11,355 bp) (Table 6).

### 3.13 Analysis of conserved motifs and structural domains of NLP families of representative species in plants

We explored the differences in the protein structure of the NLP gene family, specifically its motifs and structural domains, among different plant species (Supplementary Figure S2). Regarding conserved motifs, the types and positions of conserved motifs in the NLP gene family of higher plants (foxtail millet, sorghum, rice, maize, and *A. thaliana*) were mostly similar, but there were some differences. For example, in group 1, the At2G43500.1 sequence lacked motif 1, and in group 2, At2G17150.1 lacked motif 6, At4G38340.1 lacked motif 12 and motif 6. Compared to most NLP gene families, the sequence Sb02G302500.1 lacked multiple conserved motifs such as motif 14, motif 12, motif 13, motif 3, and motif 6, and the positions of the conserved motifs were also different; motif 2 was located between motif 8 and motif 10, not before motif 7. In group 3, the sequence Si3G084600.1 and Zm2G176655_P01 lacked motif 10, the sequence Amscaffold00080.66 lacked motif 11 and motif 4, and the sequence Si1G094300.1, Sb04G038000.1, and Zm2G042278_P01 lacked both motif 6 and motif 10. Additionally, Zm2G042278_P01 lacked motif 3. In group 3 of lower plants (*A. trichopoda*, *S. moellendorfii*, *P. patens*, *C. reinhardtii*, *C. variabilis*, *V*.* carteri*, and *O. lucimarinus*), the conservative motif of *O. lucimarinus* lacked several conserved motifs such as motif 12, motif 7, motif 5, motif 8, motif 3, motif 6, motif 10, motif 9, and motif 15, and the position of these motifs had changed significantly. The types and positions of the conserved motifs of the NLP gene family sequences in lower plants were generally the same and consistent with most higher plants. From the perspective of structural domains, the members of the NLP gene family all contained two structural domains, RWP-RK and PB1. The RWP-RK domain consisted of motif 11, motif 15, and motif 4, while the PB1 domain contained only one conserved group of motif 1. This is an important sequence feature of the NLP gene family. The positions of RWP-RK and PB1 domains were relatively fixed. The PB1 domain was located at the *C*-terminus, and its right side was the RWP-RK domain. In particular, the Ol24740 sequence in group 1 had two PB1 domains, with the left PB1 domain containing only motif 4 and the right PB1 domain containing only motif 11. The Si6G248300.1 sequence in group 1 had an RPN1_RPN2_N domain located at the *N*-terminus and did not contain a conserved motif. The RWP-RK domain of At2G43500.1 had no motif 1. In group 3, the PB1 domain of Amscaffold00080.66 was incomplete, with only one conserved motif 15 detected, while motif 11 and motif four were missing.

Overall, the conserved motif 2 was found to be present in the protein sequences of various NLP gene family research species, and it may serve as a characteristic sequence of the functional unit of the NLP gene family.

### 3.14 Homology analysis of NLP families of representative species in plants

**FIGURE 9:**
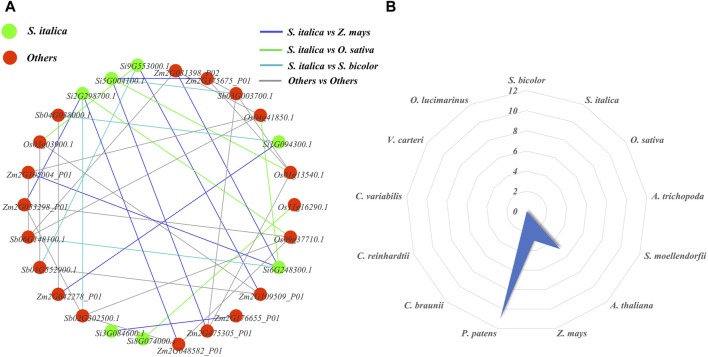
NLP family homology map and radar map. **(A)** Orthologous networks of NLP gene families in representative plants. **(B)** Paralogous homology radargram of NLP gene families in representative plants.

We constructed orthologous network relationships for the NLP families of the ten studied species and analyzed the number of paralogous gene pairs for each species using Excel statistics (Figure 9). In the orthologous network diagram, we observed nine pairs of orthologous genes between foxtail millet and maize, indicating that they had the most orthologous gene pairs compared to other species. On the other hand, maize, sorghum, and foxtail millet had relatively few orthologous gene pairs, each with only five pairs. Additionally, there were no orthologous gene pairs between foxtail millet and the remaining nine researched species (*A. thaliana*, *A. trichopoda*, *S. moellendorfii*, *P. patens*, *C. braunii*, *C. reinhardtii*, *C. variabilis*, *V. carteri*, and *O. lucimarinus*). We also analyzed the paralogous radar chart and found that *P. patens* had the most paralogous gene pairs with 11 pairs, followed by *A. thaliana* with five pairs and maize with three pairs. The remaining ten researched species did not have a pair of paralogous genes. This suggests that each of these species experienced a different scale of gene duplication events after differentiation. In ancient species, *P. patens* had the most paralogous gene pairs.

### 3.15 Expansion analysis of the NLP family of representative species in plants

**FIGURE 10:**
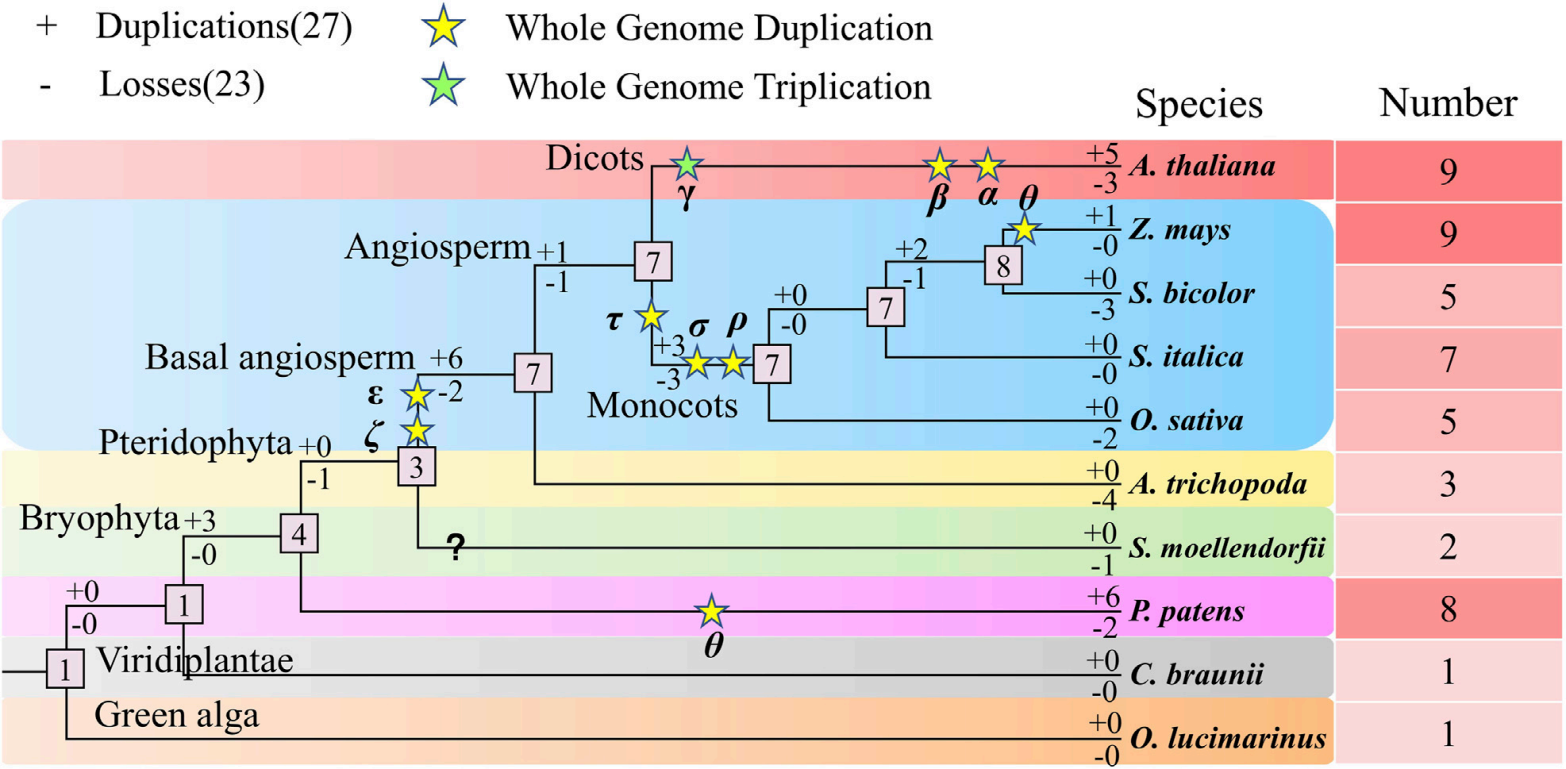
Polyploidy and amplification analysis of the NLP family in plants.

We analyzed the duplication and loss of NLP genes during evolution in the ten studied species (Figure 10). Our analysis revealed that the number of genes in the NLP family tended to expand during evolution. Initially, we calculated that the number of ancestral NLP genes in the representative species was one. The common ancestor of the representative species did not experience gene duplication and loss (0:0), and the same was true for the algae (0:0), where no duplication and loss of NLP genes occurred in both *C. braunii* and *O. lucimarinus*, with only one NLP gene present in both. Subsequently, three NLP genes were duplicated, and the common ancestor of *A. thaliana*, maize, sorghum, foxtail millet, and rice, *A. trichopoda*, *S. moellendorfii*, and *P. patens* possessed four NLP genes (3:0). After a WGD (Whole Genome Duplication) event (θ), six NLP genes were duplicated, and two NLP genes were lost (6:2), resulting in the detection of eight NLP genes in *P. patens*. Subsequently, the common ancestor of *A. thaliana*, maize, sorghum, foxtail millet, and rice, *A. trichopoda*, and *S. moellendorfii* experienced the loss of one NLP gene, creating a quantitative size of three NLP gene families. After two WGD events (ε and ζ), six NLP genes were replicated, and two NLP genes were lost, resulting in the expansion of the ancestral NLP genes in the seven angiosperms. *S. moellendorfii* experienced a loss of one NLP gene, resulting in the currently observed number of two NLP genes. For the basal angiosperm *A. trichopoda*, four NLP genes were lost, resulting in the currently observed size of three NLP gene numbers. However, ancestral species experienced one NLP gene duplication and one NLP gene loss (1:1), resulting in a sizeable number of seven NLP genes in the monocotyledonous and dicotyledonous common ancestor, with a WGT (Whole Genome Triplication, γ) and two WGD (β and α) events, five NLP genes were duplicated, and three NLP genes were lost (5:3), resulting in the currently observed NLP gene population size of nine NLP genes in *A. thaliana*. After three WGD (τ, σ, and ρ) events, three NLP genes were duplicated, and three NLP genes were lost (3:3). The number of NLP gene families in the monocotyledonous ancestor remained at seven. Subsequently, it experienced the loss of two NLP genes, resulting in the currently observed size of five NLP gene numbers in rice. The number of NLP genes in the common ancestor of maize, sorghum, and foxtail millet was maintained at seven quantitative scales of foxtail millet, with no duplication and loss occurring. However, two NLP genes were duplicated, and one NLP gene was lost (2:1), forming the number of eight NLP genes in the ancestors of maize, sorghum. With the occurrence of a WGD event (θ) in maize, after a single NLP gene duplication, the expansion was made to the currently observed size of nine NLP gene numbers in maize. In contrast, sorghum experienced the loss of three NLP genes, resulting in the currently observed number of five NLP genes. In summary, we were surprised to find an evolutionary trend of gradual expansion of the NLP gene family as a whole, with more duplications than losses throughout the expansion journey.

## 4 Discussion

Nitrogen is a major nutrient, essential to the survival of all plants (Liu et al., 2018). Therefore, nitrogen uptake and utilization directly affects plant growth and development as well as crop yields. Among the complex pathways regulating nitrate metabolism and assimilation in plants, the NLP protein family is a key component that can improve the efficiency of nitrogen utilization in plants (Castaings et al., 2009). Therefore, studying NLP family characterization and its molecular evolution in crops can provide a valuable clue to crop breeding and the evolutionary history of the family. Here, we comprehensively characterized the NLP family in foxtail millet, a C4 model crop that has not yet been resolved, then identified and investigated its molecular evolutionary trajectory by selecting representative species from each plant taxa. We obtained plenty of interesting and novel results that can be used as a resource and reference for studying NLP genes.

### 4.1 Molecular characterization and two significantly important genes of the NLP gene family in foxtail millet

We identified seven NLP genes in the foxtail millet genome, all of which were individually and separately distributed on different chromosomes. Such a small number of families implies that there may be no functional redundancy within the family members. Moreover, they were not structurally identical to each other, suggesting that these seven genes assume incompletely aligned functional roles. To the best of our knowledge, it has been shown in many studies with a large number of gene families that too many members may have functional redundancy (Wang et al., 2011; Wang et al., 2013). Thus, the small number of family sizes reflects the importance of each NLP gene. Through our analysis, we found that the foxtail millet NLP gene promoter contains a large number of light-responsive elements and hormone-responsive elements, which can enable foxtail millet to use light and related hormones for nitrogen assimilation when absorbing nitrogen. Furthermore, RNA-seq data showed that these seven NLP genes were indeed expressed in different tissues of foxtail millet and mainly in the root, which supports the known biological function of NLP genes, i.e., plants enhance nitrogen absorption and assimilation from the environment through the root via NLP proteins (Liu et al., 2022).

As the analysis deepened, we unearthed two genes that were extremely important for foxtail millet: *Si5G004100.1* and *Si6G248300.1*. The reason is as follows: we found that among the seven NLP proteins in foxtail millet, only the proteins expressed by these two genes were able to form protein-interaction networks with other proteins. For *Si5G004100.1*, only this gene maintained a stable and high expression in all four tissues (root, stem, leaves, and spica) of foxtail millet, suggesting that it plays a crucial role in the growth and development of foxtail millet. With regard to *Si6G248300.1*, its three-dimensional structure was the most unique among the seven NLP proteins, and unlike the other six members, it have almost no β-folding. The unique structure hints at its distinctive function (Ge et al., 2022). Also, only *Si6G248300.1* was involved in biometabolic processes and had the most complex and robust protein interaction network. Thus, the above general phenomena reveal the specificity and importance of these two genes. Moreover, information from the String database supported by experimental evidence showed that the protein encoded by *Si5G004100.1* interacts with the iron redox protein nitrite reductase, indicating its partial function in the nitrate signaling pathway in foxtail millet. On the other hand, Si6G248300.1 was found to interact with the proteasome subunit alpha type of the T1A family of peptidases, a multicatalytic protease complex that cleaves polypeptides with arginine, phenylalanine, tyrosine, leucine, and glutamate residues under neutral or slightly alkaline pH conditions (Bochtler et al., 1999). These results could explain to some extent how these two important genes perform their biological functions.

### 4.2 Molecular evolutionary studies reveal the origin of the foxtail millet NLP genes and the expansion of NLP genes in plants

From a duplication perspective, gene production can be traced to a variety of duplication mechanisms, such as WGD and tandem duplication (Chen et al., 2023; Feng et al., 2024). By duplication origin analysis, we found that the seven NLP genes in foxtail millet were derived from dispersed duplication (*Si1G094300.1*, *Si2G298700.1*, *Si6G248300.1*, *Si8G074000.1*, and *Si9G553000.1*) and WGD or segmental duplication (*Si3G084600.1* and *Si5G004100.1*). Deeper comparative analyses showed that the NLP family genes of sorghum and rice, close relatives of foxtail millet, were all derived from dispersed duplication, whereas the maize NLP family genes were more derived from WGD or segmental duplication (six out of nine NLP genes), which can be explained by the fact that maize underwent another separate WGD event (θ) after species formation (Wang et al., 2015). Our previous study showed that almost all NLP gene families in Brassica spp originated from WGD or segmental duplication (Chen et al., 2022a). Thus, our results suggest that NLP gene duplication origins differ significantly across plant taxa. The identification of orthologous gene pairs can help determine the origin of genes across species. The results of our analysis showed that the foxtail millet NLP family genes could form clear orthologous gene pairs with NLP family members in closely related species (maize, rice, and sorghum), although expression differences in the homologous genes indicated that their functions had diverged (Figure 3). However, it was not possible to form orthologs with NLPs from species represented in other species taxa, indicating that the foxtail millet NLP gene family may have originated from the common ancestor of monocots.

In addition to gene duplication, the evolution of gene families can be driven by a combination of factors such as base mutations and natural selection (Chen et al., 2023). Codon usage biases have been hypothesized to potentially contribute to gene evolution (Shenton et al., 2006), so we performed a comprehensive and detailed codon bias analysis. Not only were the optimal codons for NLP family of each species identified for researchers to choose from, but factors such as base mutations and natural selection were found to contribute to the evolution of NLP gene families to varying degrees. Compared with base mutations, natural selection and other factors had a more significant effect on the codon bias of the NLP gene family. Moreover, we did not detect a positive selection branch in the selection pressure analysis, suggesting that the NLP gene may be subject to purifying selection.

More deeply, we explored the expansion of the NLP gene family across the plant kingdom. We were surprised to find an evolutionary trend of gradual expansion of the NLP gene family as a whole, with more duplications than losses throughout the expansion journey. This is because all other gene families that we know about have more losses than duplications (Song et al., 2018; Chen and Ge, 2022; Ge et al., 2022; Yu et al., 2022; Zuo et al., 2022). This reflects the evolutionary particularity of NLP genes, reflects the plant’s demand for NLP genes, and also reflects the functional importance of NLP genes. Combined with the results of paralogous homology analyses, we also found the phenomenon and possible reasons for the large size of NLP gene families in the lower plant *P. patens*, i.e., each of these species experienced a different scale of gene duplication events after differentiation, whereas *P. patens* experienced the greatest number of independent gene duplications. In addition, we have also deeply compared and elucidated the differences and variations in NLP gene structure and motif sequence features of different plant taxa. Hence, we comprehensively analyzed the molecular origin of NLP genes in foxtail millet and discovered the expansion of NLP gene families in plants.

## Data Availability

The original contributions presented in the study are included in the article/Supplementary Material, further inquiries can be directed to the corresponding authors.
