# Supplementary figures and images for: Identification, molecular evolution, codon bias, and expansion analysis of NLP transcription factor family in foxtail millet (Setaria italica L.) and closely related crops
Source: Front Genet. 2024 May 21;15:1395224. doi: 10.3389/fgene.2024.1395224 (PMC11148446; doi:10.3389/fgene.2024.1395224)

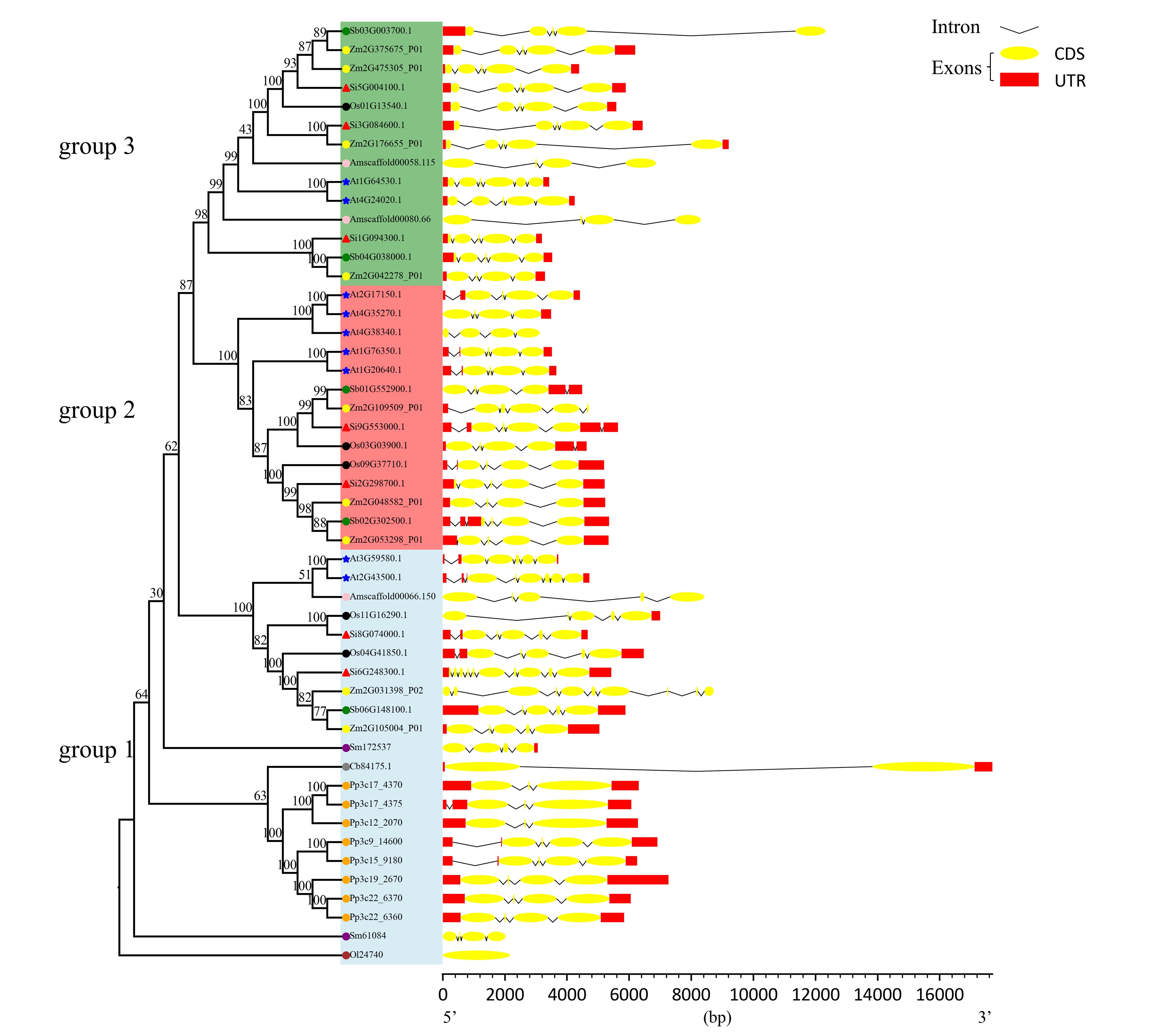

Supplement: Supplementary file 3 [file Image1.JPEG]

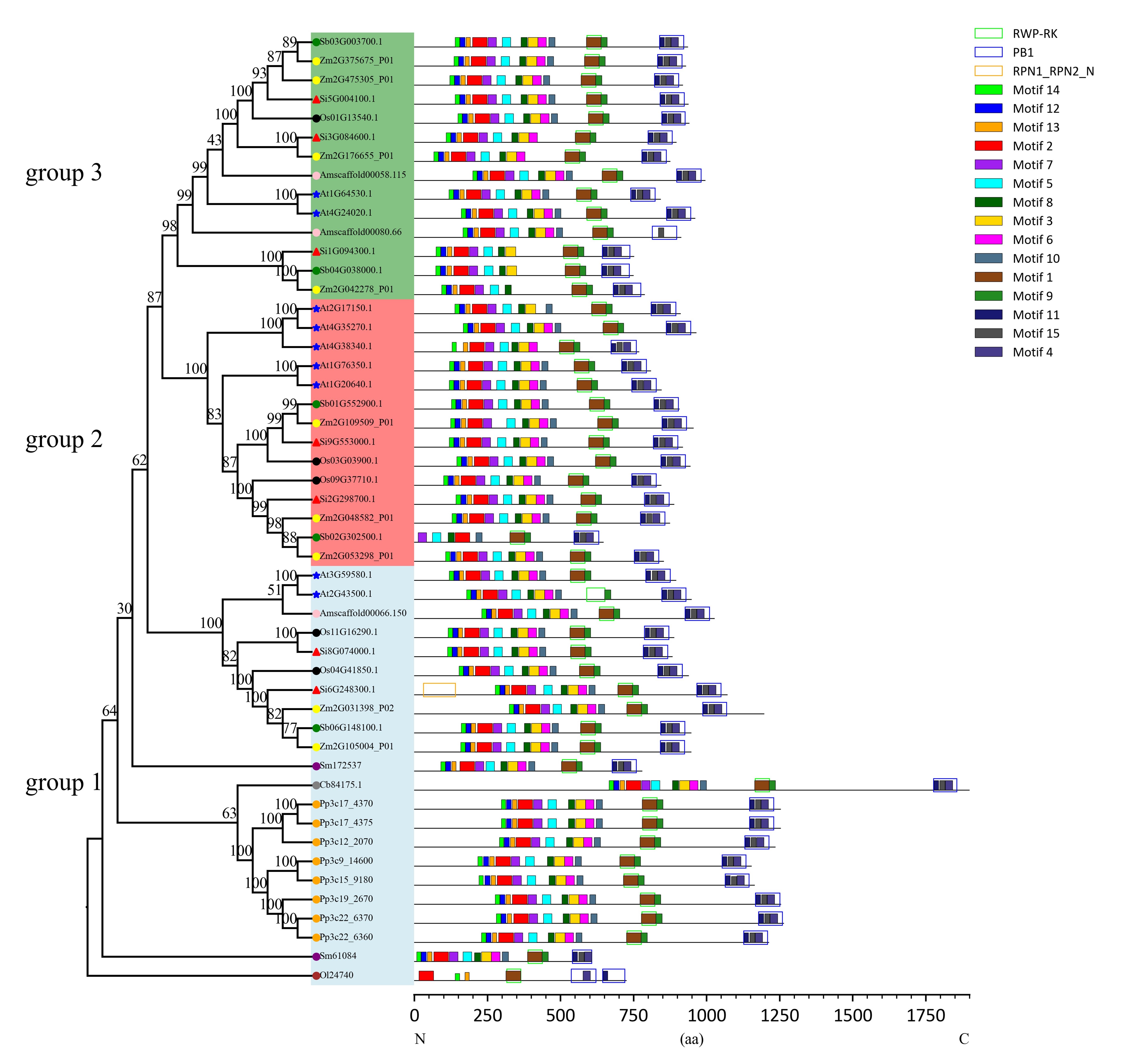

Supplement: Supplementary file 5 [file Image2.JPEG]
