# Supplementary material for: Identification, molecular evolution, codon bias, and expansion analysis of NLP transcription factor family in foxtail millet (Setaria italica L.) and closely related crops
Source: Front Genet. 2024 May 21;15:1395224. doi: 10.3389/fgene.2024.1395224 (PMC11148446; doi:10.3389/fgene.2024.1395224)
Supplement: Supplementary file 4 [file Table7.DOCX]

use strict;

open(N2O, "NLP.blast") or die "cannot open gene.family.vs.genome.blast due to $!.\n";

open(O2N, "Millet.blast") or die "cannot open genome.vs.gene.family.blast due to $!.\n";

my %new2old;

my %old2new;

while(<N2O>)

{

my @a = split(/\t/, $_);

if($new2old{$a[0]} !~ /^\w/)

{$new2old{$a[0]} = $a[1];}

next;

}

while(<O2N>)

{

my @a = split(/\t/, $_);

if($old2new{$a[0]} !~/^\w/)

{$old2new{$a[0]} = $a[1];}

next;

}

foreach my $old(keys(%old2new))

{

if($old eq $new2old{$old2new{$old}})

{

print $old."\t".$old2new{$old}."\n";

}

}
