# Supplementary material for: Identification, molecular evolution, codon bias, and expansion analysis of NLP transcription factor family in foxtail millet (Setaria italica L.) and closely related crops
Source: Front Genet. 2024 May 21;15:1395224. doi: 10.3389/fgene.2024.1395224 (PMC11148446; doi:10.3389/fgene.2024.1395224)
Supplement: Supplementary file 6 [file Table5.DOCX]

>Si5G004100.1

MDLDPPTSGPGDACSVPADAWPFDSLTTSLLFSSVSASPPLPPLPANSSSWLTPPSPLWLFEDRHLLPLEAPQAPEAAVA

AAVVEEVQRARSGNSDTTSKRVEQINPKWQFHLSLDGDGTDNSSLFKERLTQALRYFKESTDQHLLVQVWAPVKNGDRYV

LTTSGQPFVLDHQSIGLIQYRAVSMMYMFSVDGGNVGELGLPGRVYKLKVPEWTPNVQYYSSTEYPRLNHAISYNVHGTV

ALPVFDPSAQSCIAVVELIMTSKKINYACEVDKVCKALEAVNLKSTEILDHPNVQICNEGRQTALVEILEILTVVCEEHK

LPLAQTWVPCKYRSVLAHGGGLKKSCLSFDGSCMGEVCMSTSDVAFHVIDAHMWGFRDACVEHHLQRGQGVSGNAFITRK

PCFSKDIRKFCKIKYPLVHYARMFGLAGCLAICLQSSYTGHDDYILEFFLPPDCIDEDEQNALLESIFTLMKQCLRSLKV

VGDADSSWVSLQLSNVLKIENEELKTDAQFDNSDGSLNGSPEGDTHGGDHKFDNGNKKVSDMPEGHLLADDYSQDNGTSA

SRPNGSGASDSSVLHKTNKPTERRRGKAEKTISLEVLQQYFSGSLKNAAKSLGVCPTTMKRICRQHGISRWPSRKINKVN

RSLSKLKQVIESVQGSDAAFNLTSITGPLPIPVGPSSDSHNVEKVTQSKVAEPSNLAVDGDRDSSLQRSLENDGHFGIHM

AQQGFIDNNNDAQLEADKASHSRSSSGEGSINSRTSEGSCQGSPANRTFVCKPIASTFAEPQLNPEEFHKEPFQEPQLPL

SRMLIEDSGSSKDLKNLFTPAADQPFLAPPSNLVSMKHSGTVTIKASFKEDIVRFRFPCSGSVTVLKDEVAKRLRMDVGT

FDIKYLDDDHEWVKLACNADLEECMEISRNSGSHVIRLLVSDITGHFGSSCGSSG*

>Si3G084600.1

MEFDFDSPDGGDAWLLDAMATSLHFSAASPPLPPPWPCGDPHHPSVPLDTAALQDDAPGARAGKSEITKKGEPINGKCQV

HLSMVDDYSHSSYFLKEKLTLALRYFKDSTNQHLLVQVWVPIRKGDRYMLSTSGQPFVLDKRSIGLLQYRAVSIMYMFSV

DGNNVKDLGLPGRVYKQGVPEWTPNVQYYSITEYARLNHAISYNVHGTVALPVFDPSTKSCIAVVELIMTSKKVNYANEV

GKVCKALEAVSLKSTEVVEHPYVQICNEGHHAALVEMLEVLTVIGEELKLPLAQTWVPCKYQNSLVPCGGVKKSCFNIHG

SCAQELCISTSDVAFQVIDAHMWGFRDACVEHHLQKGQGVSGKAFILHRPCFCKDVTRFSKMEYPLVHYARMFGLAGCFS

VCLQSAYTGNDDYVLEFFLPPDCRKDDEQKVLLESILALLTQHLHSLHLATDEGSSEELQVSAITVINNDVQHLNFEGGI

HASHESKTNGILGPDSQKRIVSTEYEMWLSPENDTKCNGKLFVGPKGGCTSDSLLPDNNSKHQVRRRGKAEKTFSLEVIQ

HYFTGSLKNAAKSLGVCPTTMKRICRQHGISRWPSRQISKVNRSISKLKKVIESVEGSESGFTLTSITGPLPVPFSPSNP

INIKNGQQTEVIDLSIPSVQENRGSSLQSKLLENDDRLGMAIPQQSFLANLSRQIEREKASNLRSSSGEPSTHSGTSEES

CLGSPANKTFVSTLMEPQQNMWKPDSFTQELFQTQDLLLPGLFVNGSGSSENCKNHITDAVNEPSVVPLGSLMSADNSGI

VTVKARYKEDLLRFRFPCSASIIDLKDEVAKRIQADVGVFDIKYLDDDHEWVKLTCDADLEECMEISRLSGSNVLRLLVT

DIAPILGSSCGSTG*

>Si9G553000.1

MEQPAAQKDEDGLLGYAVMEDVAVGDLDLMEELFMAAPGFDFSDFSQPGPGASPGACFSPLFDICSTTTTATPPAPPGDD

DRDDTERPEARRAWLFQPRQEVEATVKERMRRALERIAQTHPGELLAQVWVPTLIGDRQVLTTCGQPFWLDSRNQRLANY

RSVSMKYQFSADESACAELGLPGRVFVGRVPEWTPDVRYFSTEEYPRVHHAQFFDIRGSVALPIFEPRSRACLGVVELVM

TTQKVNYNAEIENICSALKEVDLRSTDVSSDPHANVSLNVADTSYRAIVPEIIDVLRTVCERHELPLAQTWIPCICQAKR

GSRHSDEKFKYCVSTVDEACYIRDPNVTGFHQACSDHHLFRGEGVVGRAFGTNEPCFATDVTAYSKAQYPLSHHAKLFNL

KAAVAIRLRSIRTGSLDYVLEFFLPVDCIESEEQRAMLNSLSITIQQTCYTLRVVSLKELVDEGSFETSTVTPAEFYEKP

IHENLDEVCSNIEVPVRTTSLETSEEVTSWIASLVDAQSKGVKEMDGDLPFGFSKQEDEGFSVTAGWHTPPVLGPKGTIF

SGFKHHEEYEVKEPICSRHPSPSNLDKTVEKRRTKMEKTVSLEELRKHFAGSLKEAAKNLGVCPTTLKRICRQHGINRWP

SRKIKKVGHSLKKLQMVIDSVHGAEGTVQLSSLYENFTKTTWLERELQGDGTYPLSEQKGHLEPSVPDRQCEGRFTSHTS

GSNSMSPSCSQSSNSSHGCSSGSKSQQNGSAPQLAVKQEVFMEENQSSTLLKAASHAELQMFTEERPVTLPRSESQMLLS

EQKPVENMSGMQKSKPESLKIKAMYGEERCIFRLQPSWGFEKLKEEIVKRFSIGQEMYVDLKYLDDESEWVLLTCDADLL

ECIDVYKSSSAQTVRILVNANVQPVLGPSFGQTGLS*

>Si8G074000.1

MDGIGTSVCGAGSPPDMDSFTLSDFDGLERYTDLGAGISVDDSVLSALCSSLSSEEQAEAGASISEDGDSPDGSASGEVM

HMCTATLPKSIHGAAITLPERMLRALAMLKDASSGSGAILAQVWMPVRYGERQVLTTSDQPFLLDQRLTGYREISRKFIF

SASEGPEQFPGLPGRVFISCMPEWTSNVMYYHDSEYLRVDHAARHEVRGSLAVPVIDSSGPSDSCCAVLEVVMTQEKDNF

MSEIDSISKALQSVNLSTVKAWTYPQNLTRNQESAFTEISDVLQTVCHAHMLPLALVWVPFCSSSNANVSIEYGDQDMKF

NLRKKDLLCIHESACYANDMRMHYFVRACGDHPLERGQGVAGNVILSNSPFFSCDVKDYDVCDYPLAHHARKFGLQAAVA

IRLRSTYTGSDDYVLEYFLPLMCKGCDEQQRLLDDIAETMQRACKSLRTVSNSELMADTTVKPSNKKGCRIRFPSSDMSI

NSGHKLNVISTIKANVLSGHQKMNTNELLGDMKRAKKLKPSTTSHGEKTSTSMEKNISLSVLQKYFAGSLKDASKSLGVC

PTTLKRICRQHGISRWPSRKIKKVNRSLEKIQNVISSVHGVVDRELKYDPATGFLISSISPSGNPLLIDVEGDGVDPPHI

ESESSQLKIKLDCGASQGEYQGQLVLKAQEEKLSEIDFSLNEGRLSLNSHSSGTSNRSLSADTYNVSHFINEKATSFQTG

LGIVGSQGNNASRDLFLVPQSNIGTETLPSSSSKTDYSSGSASSHGTFQKCSKTQASANEGNTTVTVKANYKDDAVRFKL

LPSMKHHDLLEEIAKRMKLSVGSFHLKYRDDEDDWVILESDADLQECLDILEITRSHVLKVQVRDVTHPAASSGSSSVLG

M*

>Si2G298700.1

MVFLAQDVHGGMEPCLSISSRGEVKCYPDRVTCMEEGGGDPQPSFSLQARTPSEGGAAVDLDLLEQLLSGDNGWFDVVSR

SPNSLASPPPAAFFSADVTAAAVTTTSSSTQAASSWWIQTGGASPSSVRDRFSQALSYIRETQSDGDVLVQLWVPVSRGD

GRLVLTTSGQPFTLDQSSDSLIRFREVSTKYQFSADVKSGDSPGLPGRVFIGRLPEWSPDVRYFTTYEYPRVRDAQYLDV

HGTMGLPVFEKGSYNCLGVIELIMTRQKLNFTSELNTICSALQAVNLRSAEVSSVPRVQFSTASYKNALPEILEVLRAAC

LTHKLPLAQTWVTCAQQGKRGSRHSDENYPYCISTIDEACFVNEPEMQDFHDACSEHHLLRGQGVAGKAFTTNQPCFLPD

IGSSAKLDYPLSHHAKIFKLKGAVAIRLRCTRTGTADFVLEFFLPTDCQALEEQKEVLDSLSGTMRSVCQTLRVVTDKEM

EDEAMRETNELNSFTPQGKNKAEELSFGGNSADRRGEASWTSLAETSQQESELAALRMHGMFSSGGQGPSLSGVQSAAEG

SKAKRRTKAEKTVSLQVLRQYFAGSLKDAARSLGVCPTTLKRICRQHGITRWPSRKIKKVDHSLRKLQQIIDSVHGAETS

FQLNTLYKDLTNTSISSENNLSGSMTIPPTNQSNLTDFDKHQHHKSNSIVPSTSPSHSSCSHNSDSSPSCSGGARKHAPQ

GIIDLMKSGNPVKDSPIQTLQTENTSLYEHFSVHEAPTDLLQDVTEKANGGLHSSRSPSSPKQNAEANMRVKATFGSEKV

RFRLNPKCDFQELKHEIAKRLSIVDTSSLILKYLDDDSEWVLMTCDADLQECFHVYKLADIQTIKISVHLAVSPATRVTT

GPTGLS*

>Si1G094300.1

MVVGMEVEVDVDGVLQRFDFRLDRGDDGHGRGSDPCASDRSRSGGGGAEGKEVGGGGAVKERIARALRIYKEAAGDGGGA

LVQVWAPARDGGRRVLATRGQPFVLAPPQCHRLFQYRTVSLTHAFPVGGAGVPGERGLPGRVFDAGAPEWTPNVQYYGTG

EYARISYALIYDIQAALALPILEPATGSCVAVIELVTTSPRIRFADEVDKLSKALQEVALRGSEICRPAPEVCNDDAAEM

AMSEVSDILTTVGEAHKLPLAQAWVRCKRCSSSTDDHASLTAAGTPFYLADADQRLLGFREDCVEHHLRPGRGGVVEEAA

AARGPRFCADVTKYSMDVYPLAHHARFCGLAGCLAVCVQLRRDGDDASMDDSSREGCVLEFFLPLDCRDGTEQKAAADAI

AATITEHFGNGHLKATVISGLEDLSFEIDADGDCVLRPDPVIMADAPEFELNDHGGDERDSDEEGLHLAAAVGTADIEAP

KMNSGDQNGGEDPRSQVGEKKKKTKRKNEKTVSLEVLQRYFSGSLKDAARSLGVCPTTMKRICRQHGISRWPFRKIAKAN

RSLDKIKRVFESVPGSSNPMAASTPAATASHQAPAVATARGDHALPCLSSALGVASSQGSCHAPPPPPRDAALRTPLHGV

DAGVVTVKASYKGDIIRFRVPSSAGVATVKREVAKRLGLEASEFDVKYLDDDNEWVLLSCDADFQECLDVVPALSGTSTS

SGSGAAQPVIRLMVQELAEIDGSSCGSSD*

>Si6G248300.1

MAPSPPGKGKGKKKDDIDDLPEEDLALKEQLELYVLRAQDADPGVQKLALESMRQEIRSATSSMTSVPKPLKFLRPHYGA

LKSYFETMPESELKKYMADILSVLALTMSVEGERESLKYRLLGSEGDIGSWGHEYVSPMGPSDDPFGVAAMMNFDGYSEV

CSPSVADQIFSMLNDPSAAQQMFAMWSSLGSSPCTSAVREDMPFDTYSEPVDMTAAPAQRINSASALGPTGVNRELKVSD

ELVPNNGSQQGTNIIPRSVGNVLADKMLMALSLFRKSLSDGVLAQVWMPIEHNGRIVLSTFEQPFLLDQDLAGYREVSRN

FLFSVKEEPGLHLGLPGRVFISGVPEWTSSVLYYSKPEYLRMDHALRHEIRGSLAMPIYDPSKGSCCAVLELVTNKEKPD

FDAEMDSVCNALQAVNLQTATDRSSQKVPLFTSNMLHVYSENQKSAFIEILDVLRAICHAHMLPLALTWVPTSNGIDNGY

CVRNNIGVDSQSGKTVLRIHESACYINDAKMQGFLHACAERHLEKGQGIAGRALKSNLPFFSPDVREYNIEDYPLAHHAR

KFGLHAAVAIRLRSTYTGNDDYILEFFLPVNCKGCGEQQMLLNNLSSTMQRICKSLRTVSEAEADSVSASAASMNKKSNG

SLPTGNSESSSHDDQPITESAFQDLSLADKQEQDIESDQAQASSMRVAEKKRSTSEKNFSLDVLRKYFSGSLREASMSLG

VCPTTLKRICRQHGISRWPSRKINKVNRSLKKIQTVINSVHGVDRSLQYDPATGSLVPAASLPDKMPFSACDSLPTSSVG

KTMEEKSSPKSEQGFSSPDGWQRGDCQFHVSNISKREGEGDEVRMLANNNNGSRNYAPDVAKFTPHSNSEDAQGPLYPIV

VNSLRIGETGYTNSPTSLHPSIDDQTMGRNSSFVQQADVTMVDGHDTKEHTHPSTSGMTDSSSGSASSQPTFKGNPGYVI

KDRSSPALTVKATYNGDTVRFKFLPAMGWYHLLEEIAKRFRLTTGAFQLKYKDDEDEWVILASDSDLQECVDILDSIGSR

NVKLQVRDLPCLISSSGSSSCLQVTGRET*
