# Supplementary material for: Identification, molecular evolution, codon bias, and expansion analysis of NLP transcription factor family in foxtail millet (Setaria italica L.) and closely related crops
Source: Front Genet. 2024 May 21;15:1395224. doi: 10.3389/fgene.2024.1395224 (PMC11148446; doi:10.3389/fgene.2024.1395224)
Supplement: Supplementary file 9 [file Table6.DOCX]

>Si5G004100.1

ATTTTTTGGAACTTTTAGGGGTACTTTGGACTTTGCTCACTTTTAGCTGGTTTAGCTAAGTTTTAGCTAGACATCTAAAT

TTAGCCAAAGGATCAAACAGGAGCCATGTCGTGGCCTTTCCTTCGCCGGCACATTCTTCTTTCCCCTCCAGCATCCACTA

TACTACCGTACCAATACCATTATTTGGTGCTGGGCAAGTTGGCGTAGCAGCTAGCTAGCTGCTGCTCTCTTGACAGCGAT

GGAATATGCCCATTCAATTCGATCATTCATAGCGAGCTGATGGAGCTGTCTGCAGTCTGAGGTGGACGACGCGGACCTGG

TGAAGATGGATGAATCGAATTAGGAAGCTGCTAGAAGAAGATGAACAAGACTAGTCATGCATCCTATTCCTATCCTACCA

GCGCCGAGCTTGTGTATATATGAAGTGTCAATGTCATGAGCGTATATCAGGTACCTGTCGCCAAGGATTTTGTTTGTCGA

TCCCATGCCATCCAGCAACGCGTACACGAATGCCATGCTCGATCGCAGGTGGAAGCAAATAATGGATCGATAGATGTGCA

GGGAGCTCGTGGATTATGAATAATGACCATTTCATCTGAGACGACAGTACGGATCGATACGGCAGGGAAGCTAGCCGGGT

CATCACTCAGATTATTACATTACACTACTGAGATCGATATATATAGTATATAGTCAGTCGGCACCCAATTACTAGATTCA

TCGGATCCGATGGATACTCCCAAGAGCCGTTCGTTCCGTACATTCATCCATCTCGACCCACCCACTTGTATAATTCTATA

TGCCTCTCCATCGTCACACCACATCACATCCATTCATACATATGCACTGTTAGTTCATCGATCTGCAGCTTAACTTCATC

GGATCCGATGATGGTAGCCTGCAGTCGGACGATGGCGAAGCTGGCCGGCCGGCCGGCCAGCTTCAGATATGATGGACCGT

ATCCTATTCCGATTCCTACATACCACTGTACAAGAGATAGGCATAGTAGTAGGCTAGCTGCCGGTGCCGGGCGTTGCCAT

CTGAATCGGAATCTGTGCCGGAGGGAGATGCAGCTAGCTAGCGGCCGGGCGGCGGTGGAAGAAGAACATGCCGATGCGCA

CTGCCATTGGCCGATCAGATCCACGGCCAAGTGGACACCCGTCGCCATCGCCACCAGGCAGCTGGTCGGCGATAGAATAT

TACGGTAGCTGCTCCGCCCGTCCGGGATTGGAGGGGGCTAGCCGAGCCGCTGCGACGCGGCCGCCGGAGTGGGACGCGGT

AGCCGGAGCCCGGTGCGTCAACAGCGACCCCGTCCATCCATCCCATCTCCCTGCTCGATATGATCAACACGTGGTGCAGC

GCCTTCCACGGCTCTCTCCCTCCCTGCTGTGCCTGTGGACCTGTGGTGGGCCCGCCGATTATTGCTATGGCCGTGTTGGA

GTGCACCCGGACATGCCCACCATTATTTTACCTCCATAGTCCTGGCCGACTGATCGACCAAAAGTATCCGATGGTATGGT

AGCCACTTGGATAATAATCTGTGCTATAACCGATACGCGACAAAGTTGTTCAATTATCTGGGTGCTATGAACCAAGAAGT

TTTCCCTTCGTCAGGCAACTTTTATGTTTTTTCTCTTTAGACAAACCAACTTCTCGTGGATTCATAGCTCAGCAAGCATA

GCATGCACTTTCCGCTATGTTTATGCTCTTTCTTTTAGAACCACTTGTATTTACTACCTTATCTCGCCGATAAATATGCT

ACATATCTAAATGAAAATGGTAAGAACCGGCATCATGTCATTGTTCCTATGTTCTCACTTGGAGTGAGAAAGAATTTCAT

AAATCCAAACCGTAACTCATAATAGAGGGCAAGGTAGATTCCGGGAGCCCCAGAGCAATAATACTCTGGTGGATGTAGAT

GTGATTCCTGGCATTATTCATACACAGGGTCTGTGCTCGTGAGATGTGTTGGTTTTGCATTTGGCTTGGCAGATTGGCAG

>Si3G084600.1

AAAGCTTCATCAATTGAAGTATTGATCCAGAGAGTGTGCCATGAATCATATAAGCAACCATCAGAGCATTTAATAAAAAG

GAAGTGGACACAACTGCACCAATGATAAAAAAAATAGAAATATTGTTAAATTAACAAAAGGACTCCACTAATAAAAAAAA

CTTACTTCTTATCATCTTGGAGGTGAAATGTTGACACATAGTTTCTATTTGAATATATTTATAAATCATAATAAATTTAT

GAGATTATAAAAGGAACCTTCATGACAAATCTTTCACTGCATCGAAAGTAGTTAGAAAAGAAGATAATGAACGAATGGCT

AAAAATGAATGCAACGATAGCGTACCTCTTGGATGATGCAACGGTTGTGGCCAATGACCATGGCAACACTAGGCTGACCT

CTCGAAAGATGCAATGGTCATGATCGAGGCAAGGCAACATTACTCCACCAAGATGCGGCAAGGCACATCGTTTTTTATTC

CTCTTATGCACAAAAATTGAATATGTAGTCTAGTACTAACGTCTAACAATCAAGTAAGCAAGATTCAAGTTGCAAGAACA

TATGTAATCTAGTCTACTACTAACAATCAGTACGAATGTATTAGATCTATCCGAGAGAGAACTCATATAGAGTAAAGTAT

AAATATGCATATATATGTTGCAGTTCTGTAGCAAGAAAACAAGCAAAACATGATTCTCATTCAAATCATATTTGAATCTT

CACCTATGATCTGTCCCTCGAGTATTGACTGTCATGCTAGGCCCCAGATCCAATCATGATCCTATAGTTTTGCGTGAGAG

CTTTCAAGACCATAACCACAAAGAGCTCTCTAAGGAAGACATCAAAGCACAAGTCTAGCTATCGTGATAAACCCGTTATG

TAGTTCTAGTCACCATGATTAGAAAAGGCACACTAATAATCTACATCGATAATATGTTGGAATAAGCATGATTATGATAA

TATAAAATATAGAACAGGGCATATAGATCGATATTGAATCATTATACAAAGTTACATTGATATGACCAATATTCTTCACA

AAATCACCACTGACGAGATATCGACTCCTAGCTAGCTCTAAAACTTAGCATGAATCTTCATGGTAGGGTAACCACAGTGA

CTAAAATCTAGTCTAAGCTAACCTCTGAACAATCACACGGCCCTCGACTCTATGTGGATTCCATTTGGGGTCGAGGATGG

ACCTATTTATACCTTGGGGATGCCATGTCACAAATGGAAGGTACACCCCTGCTTGAAATGCACTGGCTCCACCCGGTCCA

AGGTGGTGAGCCAACTGGCAAACCTTCCTTTGGGGCACTGTACTATATGACATGGCACTAATATAGTCAAAAAGTTCAGC

ACTTAGTTGTTTGCATTTGATTCTGCACCTTATGCCATGGTGGCACAGAACGAGAACCTTTAAACAGCAGAGGATTTGAA

AATTGTTTTGCAACATAAATCTTTAATTTCTGAACTTGTCATAGATAAGATCATTTGAAAAAAAAATAATAGTCATGCTT

GATTGATGCGTGAAAATGGATATGCAGCATTGAGATTTTATAACAGTTGTAATGCTGATCAATATGCGAGAGATATTTAG

AAAAAACTGAGTATCAACTAAGTAAAAACTTACATATGCCATTAGATGCTAAATATGGTAAAAATTACTACCTCCGCCCC

AAATTATAGGTCGCTTTGAACTTTCTATATTCATAGATATTATTATGCATCTAGACATAGGGTATATTTAAGTGCATGAT

AAAATCTATGAATCTAAAAAAGCTAAAACGATCATTAATTTAGAATGGAGGAAGTAGTTGTTACCATCACCTTGATATAG

AACCATTATTTTTATCCACAAAGAAAATATATTCAAGGACTAGCAAGATGTATAGAGTGACATGGAGGGCGAATTGAGCG

TAGCTTAGCTGGTAAGATTCCTTGTGGTGGAACTTGCCCTATGTTTGTATTGTGTGATTCGCAAAAAATAGAGTGGCATG

>Si9G553000.1

CTGTATTTTACTAGCAGTGGATCCTATTAGTTGATGGCATACTTTGCTCTCAACCAATCCCTATGGGCTATGGCAATGCA

GGCTTCAGTTTGGTAGTTAAATATTTTTTTTTGAAACATAAGGTAGTTAAATATGTATAGTGGAAGTGGTACCCAAAGCT

AGCTCTGATGAAGTCTGAACCCTGGATGCCTTTTCCTCACATGAGCTTGGCTGTACTAGTTCACCAACCCCTTATTTTTG

AAAGATTCTATATGTATACTTCACCTACCAGAGTAGCAGTCTACTGTCTATCACCATGGTTAGAAGGCTAATTTCGGTAC

TAAAAGATCCCACGCCCTGGTACCAAATTAACCAACTAACAAACCACAGCTTTTTCTGGACATCTCTTCAACCAAAATTA

AGGTTTATGTCAGTATCAGACCATGATATTGTATCTGGTCCCTAAATTTTAAAAATATCAGGTCAGCCAGCAATCAGCTT

GCTTGTTCGGATTAAATTTTTTCCTTCCAAGAATAAAATATATTGAAGCTGGTTGAGAGTTGAAATATACGTACGAACCT

GTCCCGGGATTCAAGACAAACCGAGCCGGAAAATATCTATTGCTCATGTCTATGCTGCACGGATATGGATACGTGTATCG

ATATCAGGCTGATACGGATACGGGGATACGCCATTTTTCCAAAAACCCAATACGTGGATACCTTTTACTACTTTTAAAAA

TAATTAATATAATACTTATAATAGGTAAATATAAGCTTGTGATTTATATCGATTTGCACTAAGCACAAGCACACAACACC

ATTAGAGCACAACGTGAGTCAAGAGGCTACACGAGGGGGATAAAAAAAAAAGCAAGGAGGCACTGATCTGCTAGAGCAAA

AGGTGAGGGAGCAGCTCAATGTATCTGTGGCGTATCCATGATGTATCGGTGTCGGATACATATCCGATACGGATACGGCA

CCCTCCCTGACGTATCGGGGTAGCAGAGGCTCACGTGGCAAGCAGTAGTGCACACGTATACGAGTATGTGCCAGTGTGTG

CATATATAATCCTCGGAACTAGTATAATTAGTATCTGATCAATTACCTGACGTGATGCTATCTCTAAGATACGAACTACT

AGCTAGTGGCAACATTGCGTTGGTTCCATGGGCTCCTGCAAAAATTCCACGCAAAGAGACAGCGTTGAGAAGACGAAAGA

TTTCCGTCTAAAGAATATTAGTGTCGTCTTCATCAACGCTGTGCTAGCTAAAGCAACAAGTTCAAGATATTCTTTGCCAA

ATGAAAATGCCCTGAGCCAATTTCTGTCTGCTAGAATCAGCGACCGTACGTTCCAGGACTCCAAATAGGCGTTTGGTCAA

CTGTACTCCATAGGCGGCTCATATTTTTTTTTTTGCGAGAGCTAGGAGGCTCATTTCTCTCGTGAGCCCCACCAATTTTG

GACCAGAATATATAGTATCAACATAAGGCCACAAAAGCGTACCGTGACGTGTACGGTTTAATTTTAATAAGTGCGTTTGA

GGTCAAATTTACACATAATTTCCCTGTTTCAAATCGTTTTTGAGAAAAGTTCTTTTTTTCTTGAGGGACTTTTTAGAAAA

GGGAAACATAACCAAAATTTTTGCGCATCTGAGAGAAACGACGTCAGGCAGGCGCGGTAGAATCTTTGGTCGGCCACGGC

CCGCACCCAATCACCAATCACCCAACCCAACCCAAAGCAGCAGATGACATCCGCACGCCGCACGGGCCGTCGCCACGGCG

TCTGCCAGCGCATGACGGCGGGTCCCGACCTGCGCATACATATCCTCCTGACCACCATCCACGTCATCCCCCCGTGAAAT

CCCGGGCCTGCCCCTCGCGGGGCCCACGGGAATCCGGTTGACCCTGCTGCCCCAACCCGCCAATGCGTGGTCCCCACCCG

GGCAAGCTGGCGGTGAGGAGATTCCGTCGTCATCGTGCGGCTGCACAAACAAAGATGCAGGCGCAGAAATAGGCCACTCC

>Si8G074000.1

AAACCACCCCCACCGGTGGGTTTTGAACGCTGGGAAATGAGGTGCTTCTATCCCAATCCGGCGCCTCCTCCATTTCCCTA

TCCATGTTTCCAAATAAAGAATTAGTTTTATTTCCCTATCCCAATCTTCATTTCCATATATCCAAACACCACCGTAGCGG

GGTACATGCTTAAAGAATAACTATGTTGTCACCCAATCACTATAATCACAAATTATAATCTAATGGGATCATGTTTCTTA

CAAACTTGTATATTGTGGGATATAAAATTGGTGCTATTATATCCGTACTTAAAAATGGTTTCCTACAATAATTAATAATT

ATACTTTTGATGTCATGTTACAAGAGATAGCAGTGAAAATGTACATTATCTGGATTTCATGCTAAGTTTCACTACTCTCA

GACCATTTTCGATGGGAGTCTTATCCTTATTAAACAGAGTGCTATATCGGTACTTTTAATGGTGATAAAGAGTTAACAAA

GAGAGAGGTGGAATAAATTTCACTATAAATTAAATATTTTCTTACTTATATGCATTTTAGAACTAGAAATACGAAATCCT

TCCCCTTAGAGCAACTCCAAGAGGCTGCTAATCTTACCCCAATACTTTTTTAGGAAAAAAAGAGAAAAAAGAACCCCAAC

AGTCCACCCAAACCTTCCCTAATTTTTTAGCAACACTAAAAAATAGCCTACCACCGCGTATATTTTAGCGTTGGCATTCC

TCCCTCAATCCTGATTCCCGCACGCTCGCGCGTCCCTTCCGATGGGCCCAATACGATGACGTGGCCTGTTTATAATATTG

TGGGCTATTTATTAGCAATCTGCTGTGGATTGATACGTTTTTGGAGGAAATTTTTTTTGGAAGAACTCCCAATACATAGG

CAGTGTTTGGTTGGGCTGTGGCTTTTGGAAAAGCTGCTGTGAGCTGTGGAAAAGCAACTGTGAGAAAGCAGCTGTGGAAA

AAGCAGAAGACCGTTTGGTTAGAGGAGCTGTGAAACTGTAGGCTATGTAAGAAATACCTGTAATGTCCCTAAAGGTTTGT

GAGTGTGTTTATATGCAAATTGCTCGTAACAAAATAAAGTTTATATATTTTCATCCATTAAAGTGATCGGTCCACATAAA

TAGAATTATATCCATCAAAAGTTCTATACAAGGATGCCATCCTTCAACATGGATGAGCAGGGAGCGGCGGACGGGCCGGC

GGCCGGCAACCGGGGGCGACGAGCGGCGGGGTGCGGCGGCCGAGGTCGTTGGGCGGCGGTCGTCCGGGAGGCGGGGTGTG

GCGGGCGGCCGGGGGTGCCGGGAGGCAGGCCGGCGACCGAGGGCGGCGGACGACGGGCCGGCGGATCGCGGACGGCTGGC

CAGCAGCCGGCGACCCGGGGCGACGGGCGGCGGGCGGCGGCACTCGGGGTCGTCGGGCGGCGGCCGTCCGGGGGCGGGGG

TGTGGCGGGGGGCCGGGGGCGCCGGGTCGGCGGGAAGCGGATCGGCGACCGAGGACGACGGATGGCGGGCCGGCGGATGG

CGTCCGGCAGCGACGGGCGGCGGGGTGCGGCGACGGGCCGCCGATGGCGGCGCGCAAAGAGACGGAAGGGTATGCGGGGA

GGAGCGAACGGACGTGTCGGGAGAAATAGATCGGGGCAAAAAACAAATCGTTACCTTCATAATCAGTGGTAGGTGGGTAA

TTTTTTACCATAAAAGCACTTGAAAGCAGGGGGAAGGTGCTTTTTGATTTTGTACTAGAGCAAAAGTAGCTTTTGGGCAA

AAACACGTAGAGCTTTTAGACCTTTTGGTTGGCTTTTGACTTTTGCAAAAGCAAAAGCAGGTTGGAAAGTCCAACCAAAG

GCACCCATAATTGTGGGGAAGAATTTTTTTTAGTACTCTTGGAGTTGCTCTTAAATCGACTGATGGCAAATATACTGATG

CTGTGGGCCCCATAGACCACGATGCTATCGAGGTTATAAAAAGCACAGTTAAGGTTTTGCTCCTGCTTCATTCGGTTCTC

>Si2G298700.1

TAATATAGAAGAGGGAAAGCAAGCAGAGAAAAGTGGGCACTCCATTTGTTTTATTTTTGGGTGGTGTCATGGTACATCAC

AGGCGAGCATTCTTGGGTGCCTTGCACTTTATCATTGATGTCACTCTCCAACTAGTGTCTTCTACTCTTCATCCAATTGA

GCCAAGCGATCTGCATGGAGCCACAAACAAAGTAGCATAAACAACCACATGATGGCCTGTCACTACAAAACAGATTCGCT

CAGAAGGAATACAAAAGGTAAAGCTGCTTTTCCATGCTAGATTAAATCAGAATGACAAGTTAAGACCAAATTGTAGGCTG

TAATCTTGATGTGTGATTTAACCATACCATTTTCTTAAAAACAAAGTCATAATGCTAGAAGCGCGTTTCAAAATAAAAAT

GTTCAAAGTTTATACAGAAAAGCAGGCCTATATTACAATATTACACCTGTACTGTGCTGGCTTGTTGCATCGTCAAGAGG

CACCAAGATTCCTGTACATGTCATGGAATAATGAATCAAAGCCTATTTTAACGCATTCCTCCCTCATCAGGGCGCAGAAA

CATGTTCACATGGGAGGAATCAAGCAAAATGCAGTTAGTACAAATAGTAAGTTACATCTTGAAAATATTTAAAGCACTTC

CAAAACAGGAACAGCCTTGGGAATAAAAATCTATTGCTCCATCATATGATTAACCTTTCAAATTGGACTTTGGTATCCGG

ATCACATGTATCTCCACCCAAACCACAATTATGTTGCTAGTTTATTGATCTCTGCTCACTAGAGCGTTGGAATAACTTGT

ACTTTTTAACCTCCCTGAGTAAAGAACTACAGAAAATAAATAAAAGTAAAGATCAAAGATGATTAAATTAAAAGACCAGC

CAATCACAAATAGACAAGTACATATCTGGCCGTACATATGTAGCTTTACGAGATGCAATCCTAGGAGCATGCACAAATCA

TATTTAAATAAGCAAGTACTTCTATGATCGCTAGGAATATTGTTGAGCTCACAAAGCGTGTAGCGTTAAAATAAATATGG

TGCAACACTCATAATTGTATCATGATTTAGTATGAACTTTTATCATTTTACCTCTCCTTTTACCTGAAGTGTCGAAGCAT

TTTTACATAAACATTTGCAAACAGTTGTTCCTTTTAGCTTGGAGATATTTAGAAGCAAACGAGATGACTGATGGGAATGC

AACTGTAAATGTAATCATTCATTTGTTTCAAAGTAAAAATGTAATCGTTCATGGTGAAGAGAATTCTAGCCCTCTGATTT

TTTTTTCTCTGCTGGATTCTGCTGTTAACAGTCATATATATGGTGGTGGGAACATTACATGCTAAAACTCCTACCTCTTT

TGAAGTAGTTGTCCCTGAATCTGTGGTTTGCCGTTCTTTGCGTAGAGAGGACAAATTTTGGTTGGCCCTACACCAATATA

TGCGAGAATCTTCAATCAGTACGCCACCCCTGATCCCCCTCTATGTTGTTACCATTGAGGTGAGACCTGTTGCTTGCATT

TTAGTGGTCTTGTTTGTGGTCCACCAGAACCAAAATTGACAAATGAATGTGTTAGCTATCAGCAATAGTTGCAACAAGTA

CAGGTAACTTTTTTGTACACATTCCTAAATTTTACACTAGATAACACAACATGAATAGACAGTTTGGGAATCATAGAGCA

ACAGGTAAATGGTTTGCCTGAAAAATAGCCTCCCCCAGTTCCACCCCACCTTTCAAAACACTCATGTTTGTTTTGTTTTT

TTAAAAATCTCAACCACCCATGGTATTGTGAAAGAGAGGATTCGTACCAAGGAATCCTTCTCAAGCTGAATAATTACCCC

CTGCAAGTTGACTTTAATATGGAAGCGGGTATGGTCGGATTCCTGCGTGGTGCAGCTGGGCCCCCCATGAGAGGGAAAGA

TTCTAACAAGTAAAGAACACAAAATTGCAGCGGTGGCCCCACAAGCTTTGTTGCTACGACACAACCAGAGAAAGGAGGGC

>Si1G094300.1

CTGTGTGTTGGGCAGACCAAGTTGCCAGTAGATGGGCCAAACCAAGTTTAATTCAATGTATCTATGCATTCTAGAAAGGT

AGTGGTGGTGCTGGCGTGCTGCATTCGGTGGTTTCACGGTCACTCAACAAATCGAGAACATAAATTACGTGATCTTGTTA

AGAATAGCTCGTCCATCTCAAAATAGGAGCGTCTACGATAGAATATTTTATGCTATCTGTAAACTCACTCGGTTAGTATT

TTTTTTTTGAAAATTAATAATACTATTCGTCGAGTCTCTTGGAGATACTTATATGAACATACTGTGGCAGTGGGACGCAA

GAGACAAGACCCCCTCCCCCAACCATGCAAATTTCCACTACAAAATTCAAATTTTAACTAAATTTTTGAAAATTTTAACG

CTCTCCTCCCCCTAATAAGGTAAAATTTGAGTTTCTTGTTCCACCATTAGCTACGGTTGTATATGTGTATTCATATAGAT

CAATGTGGATCTCTCGAAGATGCTTATAGGGAAGGATTACACACATGTGTTTATAATGATGTGTGTCCCAATCTCTTGGA

GTTCTTCAAAACAATATGATTCACATGTAATAAATACGGACTCTTCATGCTTCATATGTAGTAGTTTTATGTTGGCATCT

CATAACTAGGTGAGTAGCATTGTTGAAGCTACTTGTGGTCTGACTTGGTACGCTCTTCAAAAATAATTTTTACTCGTAAT

TCATCGCATACTAGTAGTGTTTATAGCATGAAAATAATAACCACCTATTTTCTTTAATTCAATCCAATGATATTATTTAT

AAAAAACAAAATTTACATTATTCAAGACTATTTATTGATAAAAAATAAAAATTTGAATCTTGAAGTTCGTGTGTGTACAC

GTCTCTTATATTGGTTTAGTACTGGATGGGGGGAGAAAACATCTGAAAGTGTTTCACCCGGTACCATCTCATATAAAAGG

CAAAAGAGGACTTGTCATGTGCATTAACAGTTGGGTTACAGCCTATAGCTGGCTACTGGTCTGTTCAAAAAGCCAATGAG

AACTGACTAGGATGCGCCGCTGAAACTGAAATGGCAGCGATAACGATCTGAAAAGCGCTTTGCCCGCCGCTCATGTCGAC

CCGACCCGAGTCTATCCACGCACCGAAGCTGTGACGAACTTGTCCCGCCATCCAGGGTATGTGAGCGCCGTGCTGTACCT

GTCCGCGTTTGAGCACAGAGTGCAAGGTCTGACGTGGACCCATACCTGTTTTAATCCCTGTCAAGTGCTAACGTTGCGCT

TACTCAAATTCGTCTAACCGCATTTCAAAACCAAGTGCAGCTCCTCTTCCACGACAAACTCAATTTGATGGCTAATGGCA

CAGCATTTTCTCCCCACCTACTTCAAAGGTACTATCCACACCCAATCACTTCCGAGTTCCAAATCCTTATGAAAAAAAAT

TCCCTCAAATTGTAGATCGTTTTTGTTTTTCCAAATTCATAAATATTATTATGCATCTAGATCTACACTAGGTGCATAGT

AAAAATTATGGAATGGAAGAAGTATTACTTTAAATATGAAGAGAAAAGAACTCAATCCGAATAGAAGATCTACATTGAAG

TATTCTTTTTACTCAACAGGCATGTACTTGTGTCTATTGAGTAGTGAGCGCCAACCTATTCTTACCTTGTTGGTTCTTCT

TTCCTATAAGAGAAACACACTAGCCTAGTCACCTTTGTCGTGGTCGTTCTTTTTTTATAATAATTATATAATATACCAAA

ATAATCCAAAGAGTTTTTTTTATCGTACTTGCTCCAGCACCCCAATGCAACAAGATGTGAGATGCAAATTGCAAAAGTCC

AAAGCCGGAGTGGCACGAAGATAAGCTTGTTTAATCGCCCAAGGGATTAGTTTAGCTGCGGATTAGCCCGGTCCGGTCCA

AACCCAACCCCCAACCGGCATATTCCGTGGATCCCCGCGCGGGCCTCCAATGGCGGCCCGCCAGCTGGCGCCCCCCACTT

>Si6G248300.1

TTTTCTATCAGATTTTTTTTAAGAAAAGGTTAATCAGTAGTAGTACGAGTAGTCGAATATATATATATATATATATATAG

ACGTACAGTGGACGGCGGTTTCTCAGCATGTTGCTTTTAGCTTAATTTGGCTTCTTCTTATTACTTAGTACTCCCTCGGT

TCCAAATTGTATGTCGTTTTGACTTTTTTAATTCATAGATACTATTATGCACCTACACATGCACTATATCTAGATGCATA

ATAATATTTATGAACTAGAAAAGCTAAAACAACATACATTTTGAAACAGAGGAGTAGCTCAAAGCAGCTTTGAATTGTTA

TCCAACTCTTCCTTTTATTTGTATTTCGAATCATCATGCACATAGTAATGCATGTACCTACGTGCTCCACGCACGGTCGG

AACAGAGCGGGAGGCTTTTTTTTCTATACTCATCCTGTTCCCCTCTTATCGCCAACCATACGGACATGCCACTCTTTCAG

CATGTTGAGCTTCAGAAGTTGGTTGTATACACATCAAGTGCCTGGTTTTGGCTCACAAGTATTTGCAAGCATGAAAACTA

GTTGAATGATCAACCGAAGGAAAGAGAGAGTTCATTGGTGCACGTTTCGTCCACTTCTGAAGTTTCAAGATGCTTAGCTT

GATATATACTACTACCGAACTGATCAGACAAAAAGGGTAACACTGTGGGTACTAAAAAGTACAGGGCTACCGGTCTACTG

AAATTGGATTTTATATTCTGCCGCGTCGATCGATCAGGTACGTACCACCAGTACCACGGGCCGCAGCTTTGATGCTGTAC

TGCAGCCTGCATCTTCAAAAGGATTTCTAGTCCTTTGTGCTGACTTGTCTGATACACTTTACTGTAGCTTCCCATGTAGC

CTTCCACACAGGACATGGGATGCATTTGTTAGCACTGAAATTAACTAAGGATTCAAGCCATTTTAATTGTAGTACCAATA

CCTTAAAGCTGCCTTAAAGTAGGTTTTTTGAATGCTAAACTAACAACTTTTATTTTATTTAGAGAGGGAGGGAGTGGGGG

GGGGGGGGTAGGAAAGAAACAAAACCAACAATTAGGGGTGAATAACTCCAAGCCTAGAGCTGCCACGGCTTCGTCCGGCC

GAGCAATAGGACCAAAAGATTCCGTTAAAATTTACGGGAAAAAAACGAACATAGGGTCCATCGTCCCACAGTAAGGCCCA

TCGGTGTGTCCGTCCGTGTGTGGTGGCTTCATTGGTTGGGAGGAATCATGGCGGAAGAAAAAAAAATCCATGCGCATCGG

TGCATGCATGGTAACATGCATCAAAGAAAGGCACTAAGCAACTTGTAAAAAGCTTTGGGCTTGGATCCTTTTCTTGCGAG

AGATTGTTATGGATATCTGTTTGCTATCCCGAAATTATGGGCGTTCTACAAATCGTTTCCTAGTTCCTACGCGTCGGGTG

ACCGAAAACAATGACAGTTGGTTGCATTTGCATGCCGATAGCAAAGTTTGCATTGCCGAACTATGGTTTGCGGTACTGGA

TGATGGCCACAGGAGCCCTGCTGGATCATGGTGGAGATGGGCGGCACAGTGTGGATACATGAAACAAGCTCTTCTGTTTT

TTGGTAGAATCTGAGAAATTTGCGTCTGAATTTTCTTCTGTTTCACTCTAGAATGTCTGTTTTGTTTGTTATGAACTCCA

CTTTCTGTAGGGAAATGGTTTGCTGAGTCCCTACCAAAACGGGTTCTATGGCGCCTAAATGATACTAATTAAAAGGGGTG

TTTTAAATTCTAGCTGTAGCTCTCTGTGTGGAAATGACCAAACTGTTTGCACCTGCCTGGACCTGTATCACCTCGAGAAA

TCTCGTTTGGCGGTTGCGGCCACAGGCACTAGTGTTAAATGCTGCATTGGGCCGCTTGCACACGCGAACTGCTATTTGGG

CTCCTTGGGCCGGCCCACTGTCTGGGTCTGTGTGGTTGTGACGGAAGCGCCGCCGCAGCAGCCAACACGCGCACGCGGAG
